# Petrology and geochemistry of olivine-phyric shergottites LAR 12095 and LAR 12240: Implications for their petrogenetic history on Mars

**DOI:** 10.1111/maps.13262

**Published:** 2019-02-21

**Authors:** Emilie T. DUNHAM, J. Brian BALTA, Meenakshi WADHWA, Thomas G. SHARP, Harry Y. MCSWEEN

**Affiliations:** 1 Center for Meteorite Studies, Arizona State University, Tempe, Arizona 8528, USA; 2 School of Earth and Space Exploration, Arizona State University, Tempe, Arizona 8528, USA; 3 Department of Geology & Geophysics, Texas A&M University, College Station, Texas 77843, USA; 4 Department of Earth and Environmental Sciences, University of Pittsburgh, Pittsburgh, Pennsylvania 15260, USA; 5 Department of Earth and Planetary Sciences, Planetary Geosciences Institute, University of Tennessee Knoxville, Knoxville, Tennessee 37996, USA

## Abstract

Larkman Nunatak (LAR) 12095 and LAR 12240 are recent olivine-phyric shergottite lnds. We report the results of petrographic and chemical analyses of these two samples to understand their petrogenesis on Mars. Based on our analyses, we suggest that these samples are likely paired and are most similar to other depleted olivine-phyric shergottites, particularly Dar al Gani (DaG) 476 and Sayh al Uhaymir (SaU) 005 (and samples paired with those). The olivine megacryst cores in LAR 12095 and LAR 12240 are not in equilibrium with the groundmass olivines. We infer that these megacrysts are phenocrysts and their major element compositions have been homogenized by diffusion (the cores of the olivine megacrysts have Mg# ~70, whereas megacryst rims and groundmass olivines typically have Mg# ~58–60). The rare earth element (REE) microdistributions in the various phases (olivine, low- and high-Ca pyroxene, maskelynite, and merrillite) in both samples are similar and support the likelihood that these two shergottites are indeed paired. The calculated parent melt (i.e., in equilibrium with the low-Ca pyroxene, which is one of the earliest formed REE-bearing minerals) has an REE pattern parallel to that of melt in equilibrium with merrillite (i.e., one of the last-formed minerals). This suggests that the LAR 12095/12240 paired shergottites represent the product of closed-system fractional crystallization following magma emplacement and crystal accumulation. Utilizing the europium oxybarometer, we estimate that the magmatic oxygen fugacity early in the crystallization sequence was ~IW. Finally, petrographic evidence indicates that LAR 12095/12240 experienced extensive shock prior to being ejected from Mars.

## INTRODUCTION

At present, there are 123 known distinct Martian meteorites totaling a mass of ~140 kg (NASA 2018). Because these are the only samples we have from Mars, and are predominantly of igneous origin, they are crucial to our understanding of the magmatic evolution of the red planet. Shergottites (presently totaling 108 distinct samples) are the largest group of Martian meteorites and, based on their modal mineralogy and petrography, they are categorized into basaltic, olivine-phyric, and poikilitic classes (e.g., Goodrich 2002). Moreover, based on their minor and trace element abundances, initial Sr, Nd, Hf, and Pb isotope compositions and crystallization ages, the shergottites are categorized into enriched, intermediate, and depleted groups (e.g., Symes et al. 2008). Understanding the processes that affected these Martian meteorites during their petrogenesis can provide insights into the magmatic evolution of Mars.

The Larkman Nunatak (LAR) 12095 and LAR 12240 samples are olivine-phyric shergottites that were found in 2012 on the Larkman Nunatak ice field in Antarctica. Initial characterization suggested that these two meteorites are likely paired, but it was noted that they are distinct from another shergottite LAR 12011 that was found that same year (Balta et al. 2015a). In this work, we report the petrology and geochemical characteristics of these shergottites to evaluate their pairing, relationship to other shergottites, and their petrogenetic history on Mars.

## ANALYTICAL METHODS

We obtained one thin section each of LAR 12095 (,22) and LAR 12240 (,16) from the Meteorite Working Group.

### Petrography and Major Element Analyses

Petrographic characterization of the thin sections was performed at the University of Tennessee. We obtained photomicrographs of each slide using a Nikon Eclipse LV100 POL polarizing microscope, and images were processed and stitched using the Nikon Imaging Software Elements package. Backscattered electron (BSE) maps of both sections were obtained using a LEO 1525 field emission scanning electron microprobe (SEM) with a Link Oxford EDS system at the University of Tennessee, with individual frames combined using Adobe Photoshop. Using the BSE images and optical imagery (processed using the ENVI software package), we determined modal abundances by isolating each phase and counting the number of pixels represented. Additional BSE imaging was done with an FEI XL30 environmental SEM in the Eyring Materials Center at Arizona State University (ASU). This field emission SEM was operated with an accelerating potential of 15 kV.

Quantitative mineral analyses were performed using the Cameca SX-100 electron probe microanalysis (EPMA) instrument at the University of Tennessee in wavelength dispersive mode, with an accelerating potential of 15 kV. A beam current of 20–30 nA with a focused beam was used to analyze olivines, pyroxenes, and oxides; a beam current of 10 nA with a 10 μm spot size was used for glasses, phosphates, and maskelynite. The ZAF correction procedure was applied to all measurements. Typical detection limits were 0.05% for oxide abundances.

One olivine grain was mapped in each sample at high EPMA current to observe zoning in minor elements. Minor elements Cr, Al, and P were mapped using 2 μm step sizes across a 1024 × 1024 μm grid using a 100 nA beam current and with counts collected for 900 ms per spot. This analysis generally followed the technique of Milman-Barris et al. (2008); however, compared to that study, lower EPMA currents were used here to limit contamination of the probe and were found to be adequate for characterizing minor element zoning.

### Crystal Size Distribution Analyses

Crystal size distribution (CSD) analysis is an effective tool for quantifying crystal growth rates and nucleation history in magmatic systems (Marsh 1988). Using the thin section images described previously, we mapped olivine boundaries as required for CSD analysis. As is typical for Martian samples (e.g., Balta et al. 2015b), we found that pyroxene grain boundaries were too poorly defined in BSE to analyze by CSD and would require techniques specifically designed to target that mineral (e.g., Lentz and McSween 2005), which was beyond the scope of this study. Olivine shapes were digitized and converted manually to length and width measurements using the ImageJ software. Olivine shape measurements were then processed using the CSD*slice* software (Morgan and Jerram 2006), which converts two-dimensional crystal sizes of random slices through grains into a three-dimensional crystal size distribution. Best matching habit ratios (short-, intermediate-, and long-axis ratio) calculated with CSD*slice* were entered in the CSD*correction* software (Higgins 2000), which generated the CSD plots. The CSD results are plotted as described by Marsh (1988) and Morgan and Jerram (2006) with crystal size plotted against number of crystals per unit size in a semilogarithmic plot, and best matching habit ratios as calculated by CSD*slice* are reported for comparison to other work.

### Trace Element Microdistribution Analyses

The abundances of rare earth elements (REE) and additional selected trace elements were measured in situ in individual grains of olivine, pyroxene (low- and high-Ca), maskelynite, and merrillite using the Cameca IMS-6f secondary ion mass spectrometer (SIMS) at Arizona State University (ASU). Measurements were conducted with an O^−^ primary beam, at currents ranging from 5 to 15 nA, and at an accelerating voltage of −12.5 kV. Typical counting times ranged from 1 to 2.5 h depending on the analyzed phase. Measurements were made using the energy filtering technique of Zinner and Crozaz (1986) and a −75 eV energy offset to avoid molecular interferences.

Concentrations of REEs and selected trace elements (Ti, Y, Zr) were determined relative to the abundance of a reference major element: Si for silicates and Ca for phosphates. Standards such as NIST glasses 610, 612, and 614, as well as basalts KL2-G and ML3G were measured at the beginning, middle, and end of analytical sessions to monitor reproducibility and instrument stability. A correction for the light rare earth element (LREE) oxide interferences on the heavy rare earth elements (HREE) was made because not all monoxide isobaric interferences are eliminated using energy filtering (Zinner and Crozaz 1986). Procedures of Zinner and Crozaz (1986) and Hinton (1990) were utilized. We measured these interferences with the ASU IMS-6f standard REE glasses, REE1, REE2, and REE3 (also known as Corning Glass X, V, and W; Drake and Weill 1972), to calculate the correction factors. Our measured MO^+^/M values are similar to the Zinner and Crozaz (1986) correction values.

### Raman Spectrometer Analyses

Raman analyses were conducted to characterize an area in LAR 12240 that had an inclusion of pyroxene composition within a pyroxene grain (described below in the Petrography section). Analyses were conducted using a custom-built Raman spectrometer at ASU in a 180° geometry. The sample was excited using a 100 mW Coherent Sapphire SF laser at a wavelength of 532 nm. The laser power was controlled using a neutral density filter wheel and an initial laser power of 150 mW. The laser was focused to a ~1 μm spot onto the sample using a 50× superlong working distance plane APO Mitutoyo objective with a numerical aperture of 0.42. The signal was discriminated from the laser excitation using a Kaiser laser band pass filter followed by an Ondax^®^ SureBlock™ ultranarrow-band notch filter and a Semrock edge filter. The data were collected using an Acton 300i spectrograph and a back-thinned Princeton Instruments liquid nitrogen cooled CCD detector. The best spectra were produced by analyzing spots at 100 μW for 5 min (60 s exposure time and 5 s accumulation) and were corrected against the spectra of a cyclohexane standard.

## RESULTS

### Petrography

**Fig. 1.**
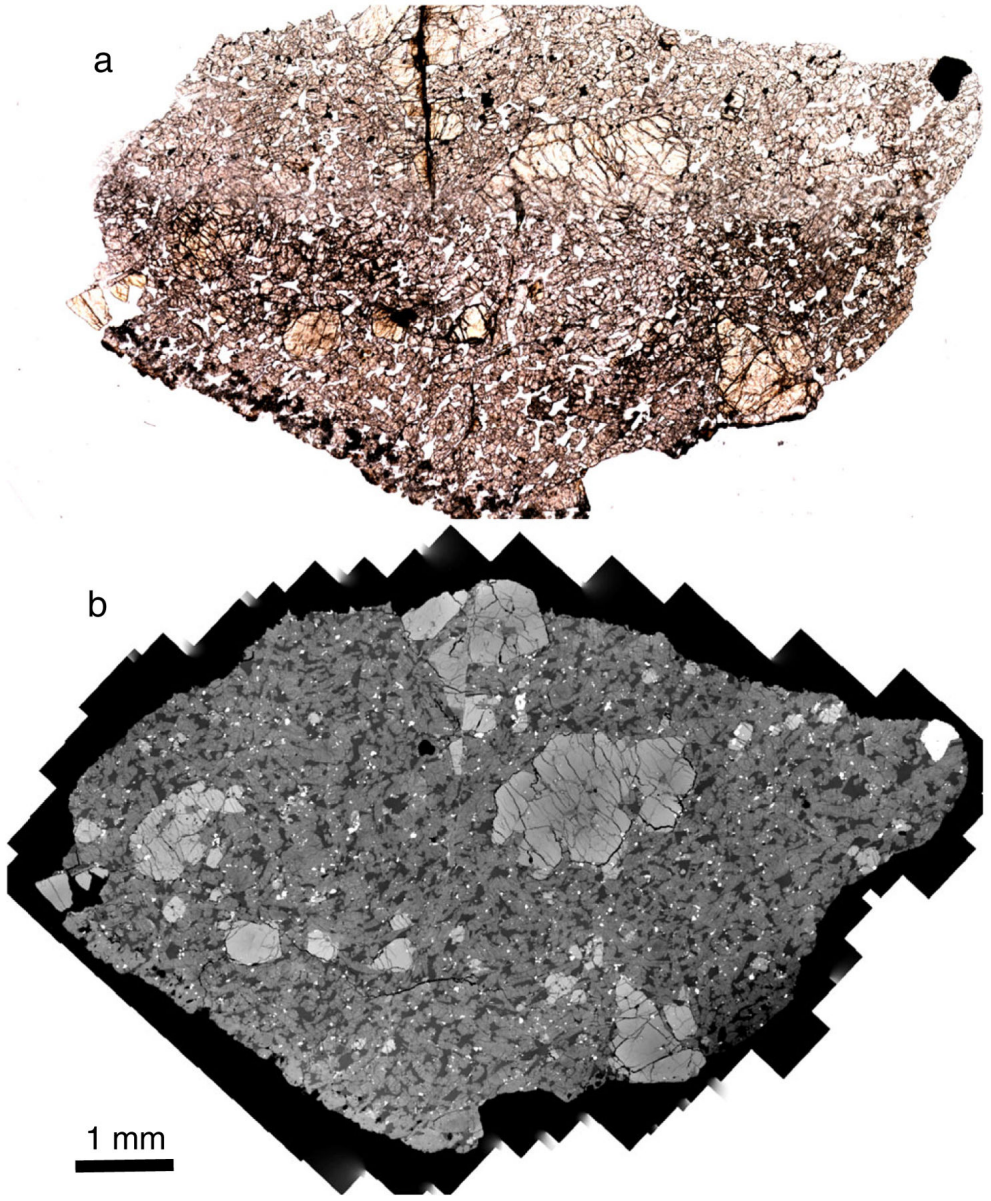
Thin section of LAR 12095,22 viewed in (a) plane-polarized, transmitted light and (b) BSE imaging. (Color figure can be viewed at wileyonlinelibrary.com.)

**Fig. 2.**
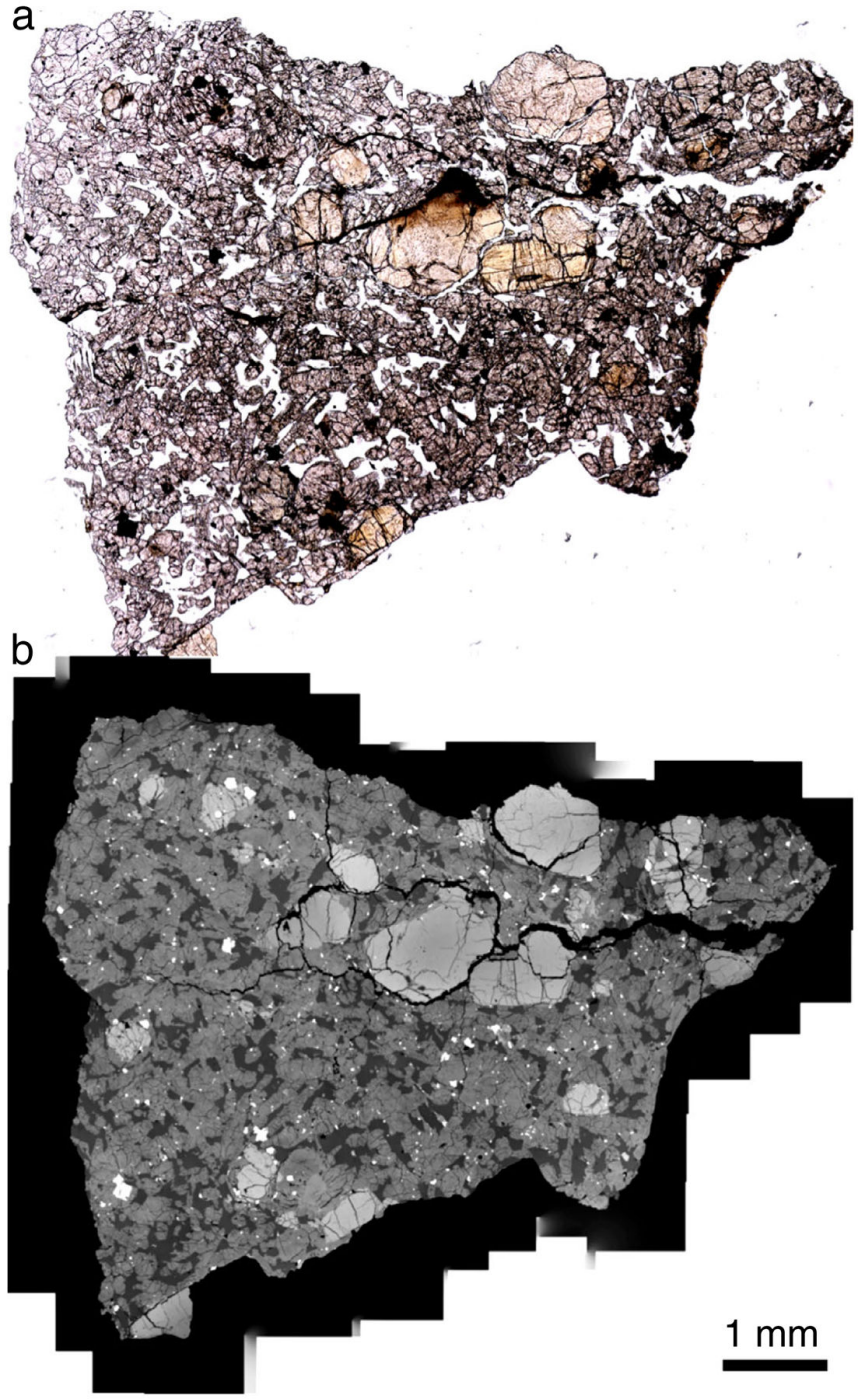
Thin section of LAR 12240,16 viewed in (a) plane-polarized, transmitted light and (b) BSE imaging. One olivine megacryst located adjacent to a shock melt vein, near the middle of the section, is a reddish-brown color in (a). (Color figure can be viewed at wileyonlinelibrary.com.)

**Fig. 3.**
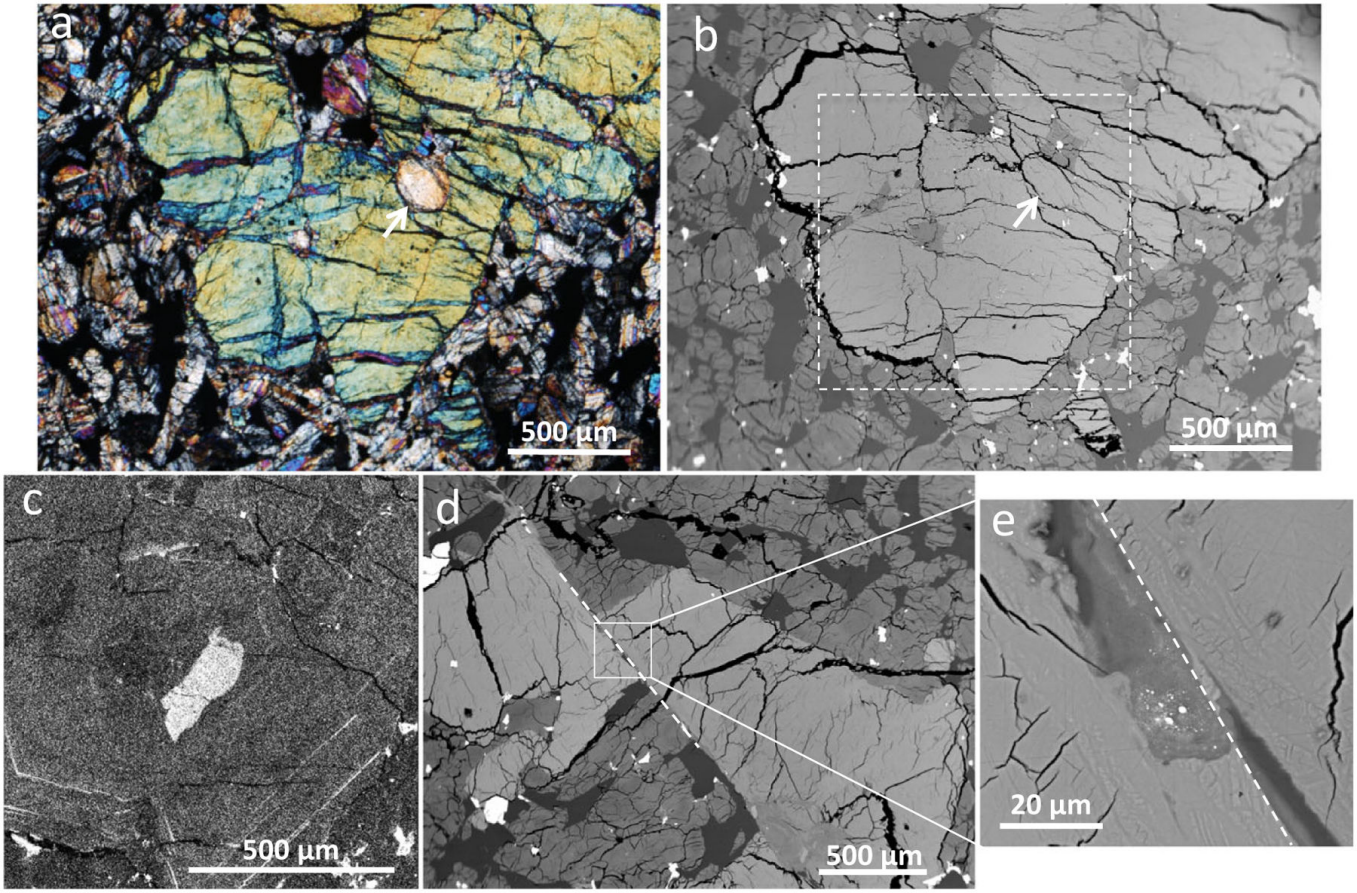
Olivine textures in LAR 12095. a) Cross-polarized, transmitted light image of olivine grains (one example indicated by a white arrow) included in an olivine megacryst area—inclusions are notably different in orientation and birefringence from surrounding olivine. b) BSE image of the same olivine megacryst as in (a); notably there is no BSE contrast between the enclosed inclusions (the white arrow indicates the same olivine inclusion that is also highlighted by an arrow in [a]) and the surrounding olivine. c) Phosphorus count map of olivine shown in the white dashed box in (b); contrast adjusted manually to best illustrate zoning. d) BSE image of an olivine that was sheared (dashed line) due to shock. e) Enlarged BSE image of the area of the shock melted region along the shear zone (dashed line) shown in (d). (Color figure can be viewed at wileyonlinelibrary.com.)

**Fig. 4.**
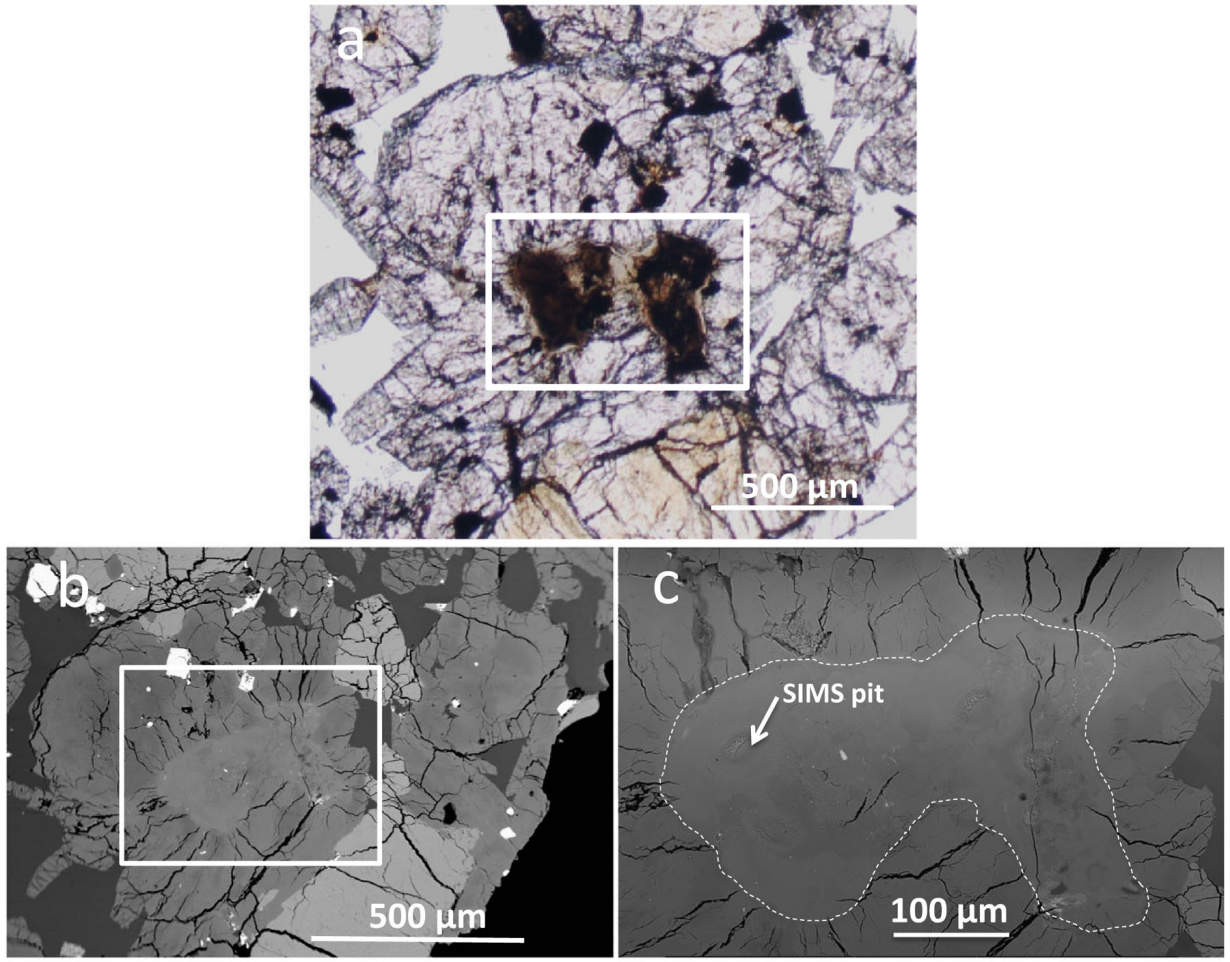
Shocked areas in LAR 12240. a) An inclusion of pyroxene composition (outlined with the white rectangle) within a larger pyroxene grain in LAR 12240 viewed in plane-polarized, transmitted light appears dark and patchy compared to the surrounding area. b) BSE image of the same inclusion (again outlined with a white rectangle) shows contrast due to the difference in major element compositions of the inclusion and the surrounding pyroxene; radiating fractures are present around the inclusion. c) A magnified BSE view of the inclusion (outlined with the white dashed line) shown in the white rectangles in (a) and (b). (Color figure can be viewed at wileyonlinelibrary.com.)

The LAR 12095 and LAR 12240 shergottites are similar in their petrographic features (Figs. 1 and 2). Both samples are olivine-phyric shergottites with porphyritic olivine crystals (up to 3 mm in length) and rare pyroxene phenocrysts (up to 2 mm in length), surrounded by a groundmass composed mainly of finer grained olivine, pyroxene, and maskelynite (0.25–0.5 mm in length), along with minor to trace amounts of phosphates, sulfides, and oxides. LAR 12095 is composed of ~17% olivine, 61% pyroxene, 21% maskelynite, and 1% spinel and sulfide grains, and LAR 12240 is composed of ~16% olivine, 60% pyroxene, 23% maskelynite, and 1% spinel and sulfide, based on the exposed areas in the thin sections studied here. Inclusions are present inside both olivine and pyroxene grains. Olivine hosts inclusions of both chromian spinel and melt, with spinel distributed throughout the grains. Dispersed micro-inclusions hypothesized to be exsolved oxides were recently reported in olivine grains in the Martian meteorite Tissint (Balta et al. 2015b; Castle and Herd 2015). Some small chromium- and aluminum-rich oxide inclusions were found in large olivine grains in LAR 12095 and LAR 12240, but in contrast to Tissint, these inclusions were clustered within grains and there were large areas free of these inclusions. The largest olivine grain in LAR 12095 contains olivine inclusions with rounded edges that are optically discontinuous with the surrounding phenocryst; however, there was no compositional difference between the inclusions and the surrounding grain (Figs. 3a and 3b). One pyroxene grain in LAR 12240 contains an inclusion of pyroxene composition that appears to be dark and patchy in visible light (Fig. 4a).

Shock features are present and most commonly expressed as shear zones that show offsets of mineral grains up to 400 μm. These zones typically have small amounts of shock melt (Figs. 3d and 3e), with shock melt pockets up to 250 μm wide. Other shock features include complete conversion of plagioclase to maskelynite, and highly fractured olivine and pyroxene grains with at least one pyroxene grain showing fractures that extend radially from a pyroxene-composition inclusion at the center of the grain (Fig. 4). Petrographic evidence of darkened olivine was observed, particularly in LAR 12240 (Fig. 2); some olivine grains in contact with shock melt are reddish-brown in color. Detailed analysis of shock effects is beyond the scope of the present study.

### Crystal Size Distribution

Crystal size distribution was performed on 199 olivine grains in the LAR 12095 thin section and 41 olivine grains in the LAR 12240 thin section; the total number of olivine grains analyzed here is low compared to those in other CSD studies of shergottites (e.g., Lentz and McSween 2005; Ennis and McSween 2014; Basu Sarbadhikari et al. 2009; Balta et al. 2015b). The number of grains analyzed here reflects the sizes of the available thin sections (particularly LAR 12240), the abundance of small olivine grains, and the area taken up by the largest olivine grains. The limited number of grains in our CSD analysis may limit the statistical significance of these results but we report them for completeness.

**Fig. 5.**
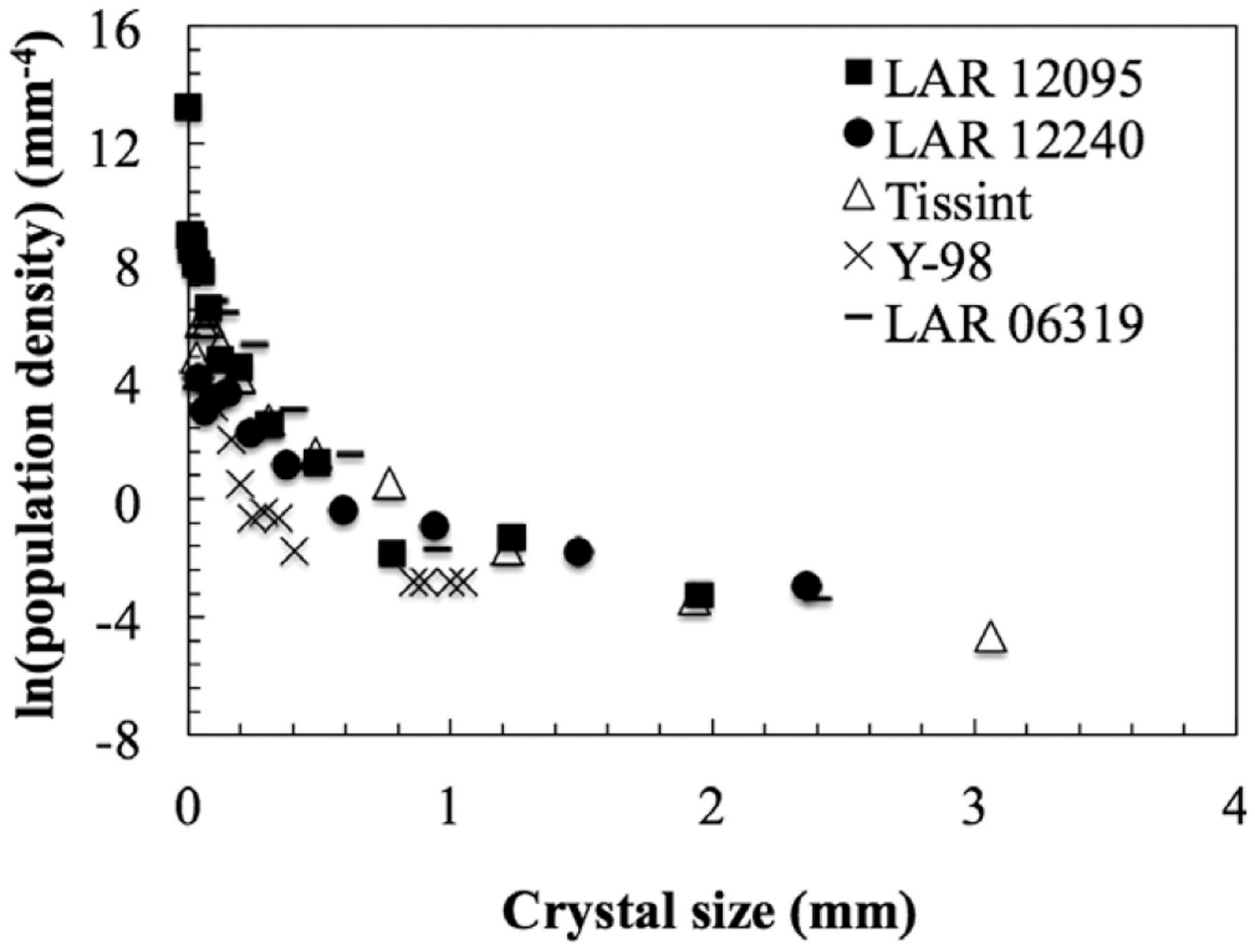
Crystal size distribution (CSD) analyses of olivines in LAR 12095 and LAR 12240. Olivine CSD data for Tissint (Balta et al. 2015b), Y-98 (Lentz and McSween 2005), and LAR 06319 (Basu Sarbadhikari et al. 2009) are shown for comparison. Crystal sizes were calculated for olivine crystal lengths in LAR 12095, LAR 12240, Tissint, and LAR 06319, and for crystal width alone in Y-98.

The best matching habit ratios determined by CSD*slice* are 1.0:1.3:2.0 for LAR 12095 and 1.0:1.4:2.6 for LAR 12240, generally suggesting similarity in olivine shapes between the two sections. Both CSD profiles show concave-up/negative slope patterns (Fig. 5) that have been previously interpreted as indicating continuous growth under steady-state conditions (e.g., Lentz and McSween 2005; Basu Sarbadhikari et al. 2009; Balta et al. 2015b). The CSD plots for both samples overlap at coarse grain sizes but LAR 12240 shows less fine-grained olivine per unit area than LAR 12095; in fact, LAR 12095 contains more fine-grained olivine than any previously characterized shergottite. For comparison, previous olivine CSD curves from Tissint, Yamato-980459 (hereafter Y-98), and LAR 06319 are also shown in Fig. 5; these samples were analyzed using similar CSD techniques to those applied here (although crystals for Y-98 were categorized using widths rather than lengths; Lentz and McSween 2005).

### Major Element Phase Compositions

Major element compositions of various phases in LAR 12095 and LAR 12240 are given in Tables 1 and 2.

#### Olivine

Olivine grains in LAR 12095 have Mg# ranging from 59 to 70, where the most Mg-rich cores are rare and only found in the largest grains (typically >500 μm diameter required). Cores of olivine grains smaller than this size have Mg# 59–64, and all olivine grains have rims of Mg# 59–60. LAR 12240 olivines have Mg# ranging from 58 to 69, with a similar distribution as observed in LAR 12095; only coarse-grained olivine (>500 μm diameter) shows Mg# higher than 62, with most grain cores being close to Mg# 60, and rim compositions being Mg# 58–60.

Minor elements (such as P) can be used to track magmatic growth of olivine (e.g., Milman-Barris et al. 2008). Therefore, we mapped the abundances of minor elements Al, Cr, and P in one olivine phenocryst from each thin section. The olivine from LAR 12240 does not show well-developed zoning in any of these elements. However, phosphorus seems enriched in cracks around inclusions in the olivine, suggesting that it may have been added to the sample during secondary alteration (Martian or terrestrial). On the other hand, an olivine grain mapped from LAR 12095 showed classic magmatic zoning, with an irregular, high-P core surrounded by a large low-P zone and high-P bands parallel to grain edges in the rim (Fig. 3c) (Milman-Barris et al. 2008). The high-P core has irregular and rounded edges and is found adjacent to a melt inclusion. The mapped area included one of the rounded olivine inclusions discussed in the Petrography section (shown in Fig. 3c) and no distinction in minor element abundances was noted across the optically observed boundary.

#### Pyroxene

**Fig. 6.**
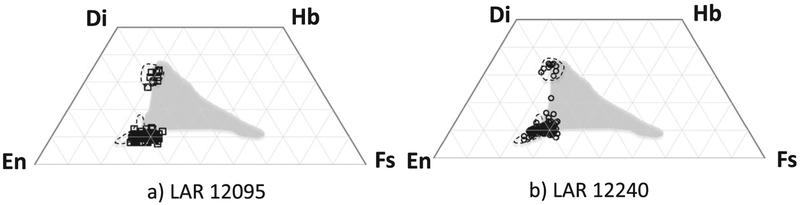
Pyroxene major element compositions measured in (a) LAR 12095 (black squares, *n* = 95) and (b) LAR 12240 (black circles, *n* = 111). Data points for LAR 12240 include analyses of the pyroxene inclusion shown in Fig. 4. Dashed fields show pyroxene compositional ranges from DaG 476/489 (Wadhwa et al. 2001); gray fields show the range of pyroxene compositions from Tissint (Balta et al. 2015b).

**Fig. 7.**
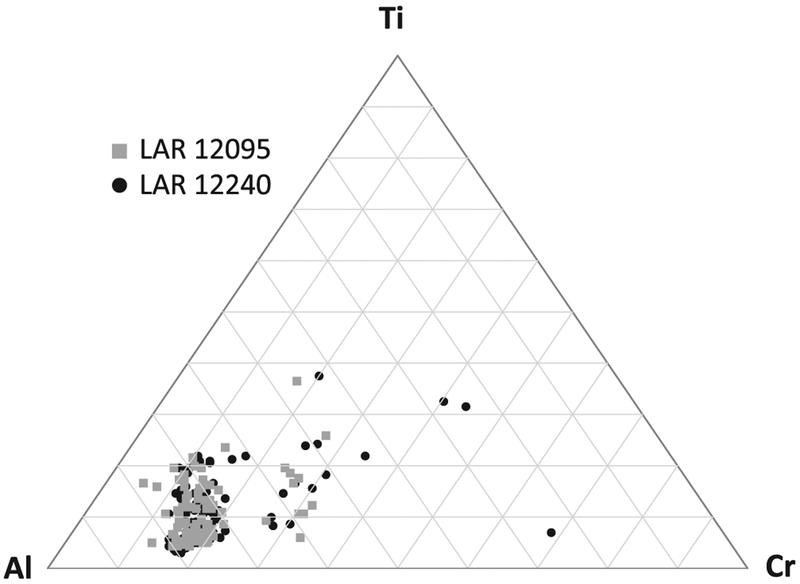
The Al, Cr, and Ti compositions of pyroxenes in LAR 12095 and LAR 12240 plotted in a ternary diagram. The total number of pyroxene analyses for each sample is indicated in the caption for Fig. 6.

**Fig. 8.**
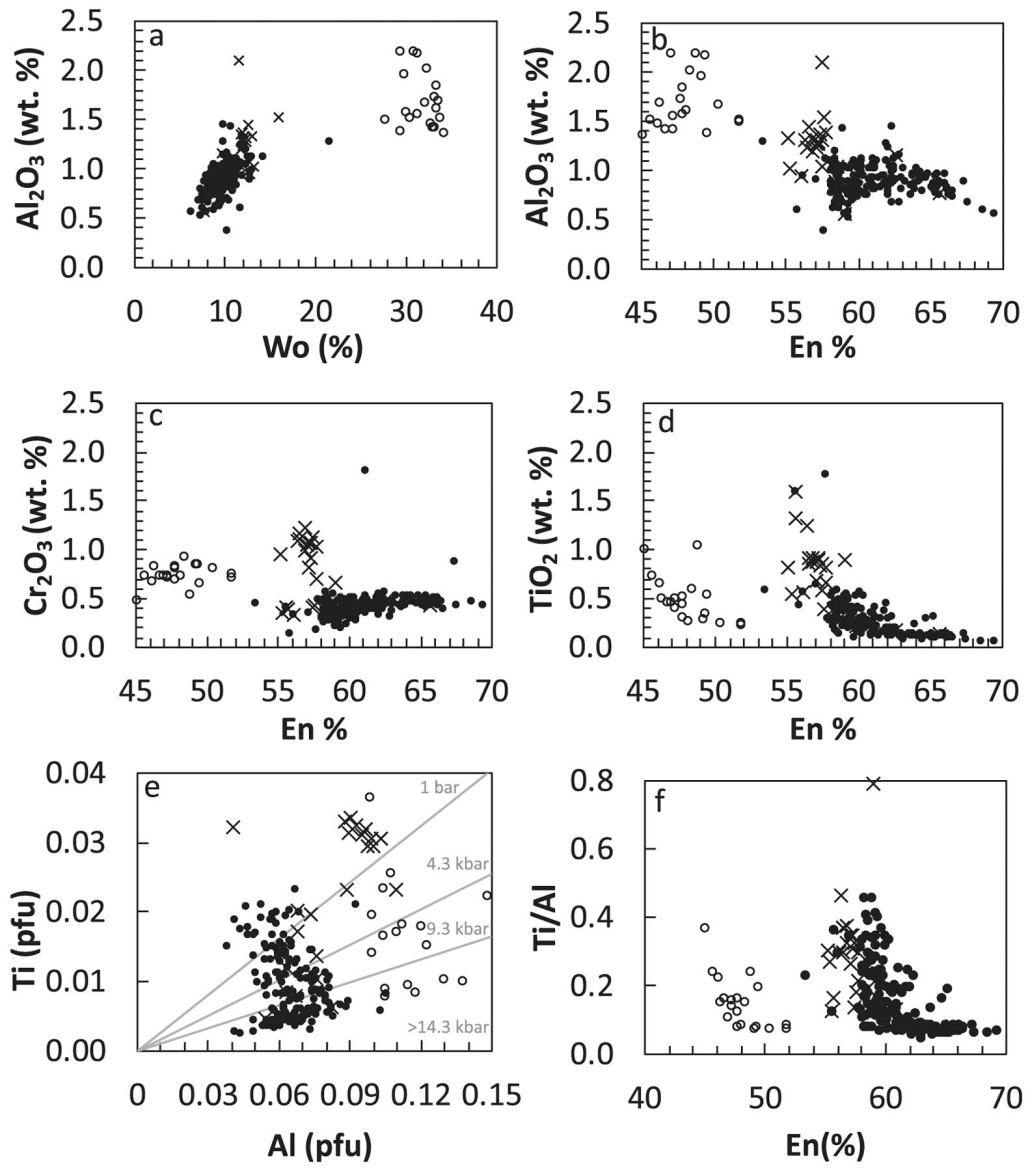
Pyroxene minor element compositions in LAR 12095 and LAR 12240. Figures show combined data from LAR 12095 and LAR 12240 since, as seen in Figs. 6 and 7, the major and minor element abundances in pyroxenes of both samples define similar compositional ranges. Pigeonites are represented by filled symbols, augites by open symbols, and crosses represent analyses on the pyroxene inclusion shown in Fig. 4. a) Al_2_O_3_ versus Wo content in pigeonites and augites. b, c, d) Al_2_O_3_, Cr_2_O_3_, and TiO_2_ plotted against En content in pigeonites and augites; as can be seen here, the pyroxene inclusion data (crosses) overlap with the low-En end of the pigeonites data. e) The amounts of Ti versus Al (per formula unit) in pigeonites and augites; gray lines and labels show pressures at which pyroxenes of these compositions crystallized in experiments by Filiberto et al. (2010). f) Ti/Al formula ratios plotted against En content in pigeonites and augites.

Pyroxene compositions (major and minor element concentrations) overlap completely in both thin sections (Figs. 6 and 7). All measured low-Ca pyroxenes are pigeonite (En_56–69_Fs_24–33_Wo_7–14_). Augite (En_47–52_Fs_18–21_ Wo_29–34_) is present but is less common than pigeonite, making up <10% of pyroxene analyses (Fig. 6). Pyroxene minor element compositions are complex compared to those observed in other olivine-phyric shergottites. Pigeonite Al_2_O_3_ contents show a strong positive correlation with their Wo contents, and a negative correlation with En contents (Figs. 8a and 8b). The Cr_2_O_3_ abundances in pigeonites show little correlation with En content (Fig. 8c). The TiO_2_ contents of pigeonites show a negative correlation with their En contents (Fig. 8d). The augite Al_2_O_3_ contents do not show a significant correlation with their Wo or their En contents (Figs. 8a and 8b). Augites are elevated in Al_2_O_3_ compared to all pigeonites but similar in their Cr_2_O_3_ and TiO_2_ contents to those observed in the more ferrous pigeonites (Figs. 8b–d). These trends are generally similar to those reported for the groundmass pyroxenes in Sayh al Uhaymir (SaU) 005 by Goodrich (2003); the broad range of pigeonite Al_2_O_3_ concentrations and positive correlation with Wo contents was interpreted in that work to result from the onset of plagioclase crystallization. However, Al_2_O_3_ and TiO_2_ contents in pyroxenes in experiments by Filiberto et al. (2010) were hypothesized by these authors to be an indicator of crystallization pressure. The lowest TiO_2_ contents in pigeonite overlap with TiO_2_ abundances in experimental pyroxenes crystallized at ~1.6 GPa (~16 kbar) for a Northwest Africa (NWA) 1068 starting composition by Filiberto et al. (2010) (Fig. 8e). The range of Ti/Al ratios in pigeonites is only slightly broader than that in augites (Fig. 8f).

The major element composition for the pyroxene inclusion in LAR 12240 described previously in the Petrography section corresponds to stoichiometric pigeonite (Fig. 6). The minor element composition of this inclusion also mostly overlaps with those of groundmass pyroxenes, including a wide range of Al and Ti contents, with the exception of a population that matches the highest Ti/Al ratio observed in the groundmass pyroxenes (Figs. 8e and 8f) but with higher absolute Al_2_O_3_ and TiO_2_ contents (Figs. 8b and 8c). There is wide scatter in pyroxene minor element compositions; however, the range of measured compositions is similar for both samples (Figs. 7 and 8).

#### Maskelynite

Plagioclase in both meteorites has been completely transformed to maskelynite by shock. Maskelynites in both thin sections overlap in their composition (An_67–53_ in LAR 12095 and An_67–53_ in LAR 12240). Maskelynites in both samples are notably low in K (Or_0.3–0.8_ in LAR 12095 and Or_0.3–1.0_ in LAR 12240). This phase shows complex zonation, with individual grains showing the full compositional range represented in each sample. Preservation of igneous zoning indicates that the maskelynite is a diaplectic glass rather than a product of shock melting.

#### Phosphates

Merrillite is the dominant phosphate in both meteorites. A single apatite was located in LAR 12095; that apatite is OH-rich, with molar OH abundance roughly 2× molar F abundance, and Cl content zoned with Cl = F at one spot and 10 × Cl = F at the other analyzed point. No steps were taken to avoid damage of this apatite during analyses, so apatite volatile abundances are approximate. Merrillites in both LAR 12095 and LAR 12240 are Mg-rich with Mg molar abundance ~5× the molar abundance of Fe. Notably, some merrillites are intergrown with fine-grained sulfur-bearing minerals. The exact mineralogy could not be determined from EPMA analyses as the beam spots were typically larger than the grain sizes of the minerals in these intergrowths. Nevertheless, the analysis points on these intergrowths show high sulfur, low phosphorus, and only small variation in calcium content, indicating that these minerals are likely calcium sulfate.

#### Sulfides and Oxides

The LAR 12095 and LAR 12240 shergottites contain oxide minerals that overlap in composition, with a trend from Cr-rich spinel (particularly in olivine-hosted inclusions) toward titanomagnetite and ulvöspinel (but without reaching that endmember). Both LAR 12095 and LAR 12240 also include rare ilmenites in the groundmass. Sulfides in both samples are mostly iron-rich pyrrhotites but include rare high-Ni grains.

### Raman Analyses of Pyroxene Inclusion

**Fig. 9.**
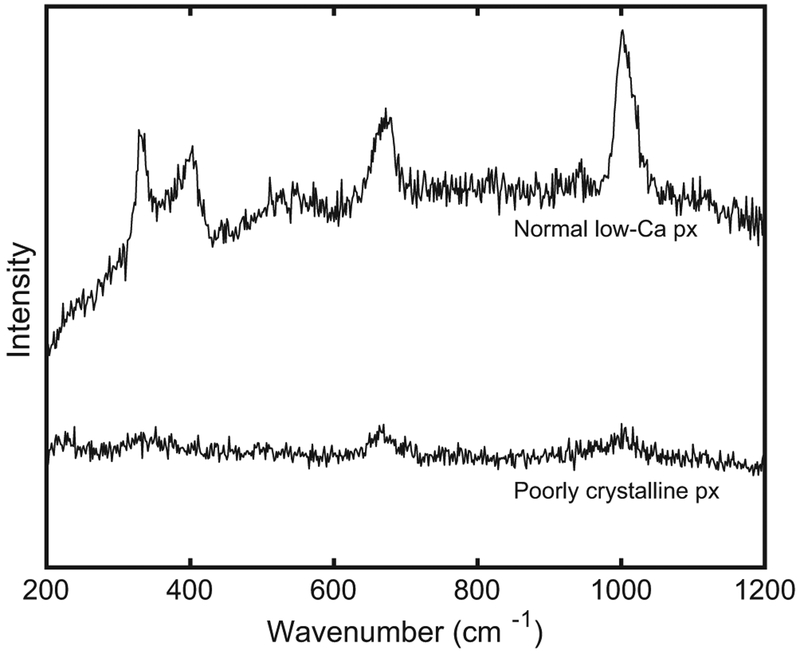
Raman spectra of the pyroxene inclusion in LAR 12240 (“poorly crystalline px”) shown in Fig. 4 and the pyroxene (“normal low-Ca px”) surrounding it.

The Raman spectrum (Fig. 9) of the included stoichiometric pigeonite within a pigeonite grain (shown in Fig. 4) has short broad peaks at 654 and 988 cm^−1^, indicating a weak pyroxene signal. In contrast, the pyroxene surrounding the inclusion displays strong characteristic pyroxene peaks.

### Rare Earth Element Compositions

We measured REE abundances in individual grains of olivine, low-Ca pyroxene (pigeonite), high-Ca pyroxene (augite), maskelynite, and merrillite in LAR 12095,22 and LAR 12240,16. Representative REE abundances in each phase are given in Table 3.

#### Olivine

**Fig. 10.**
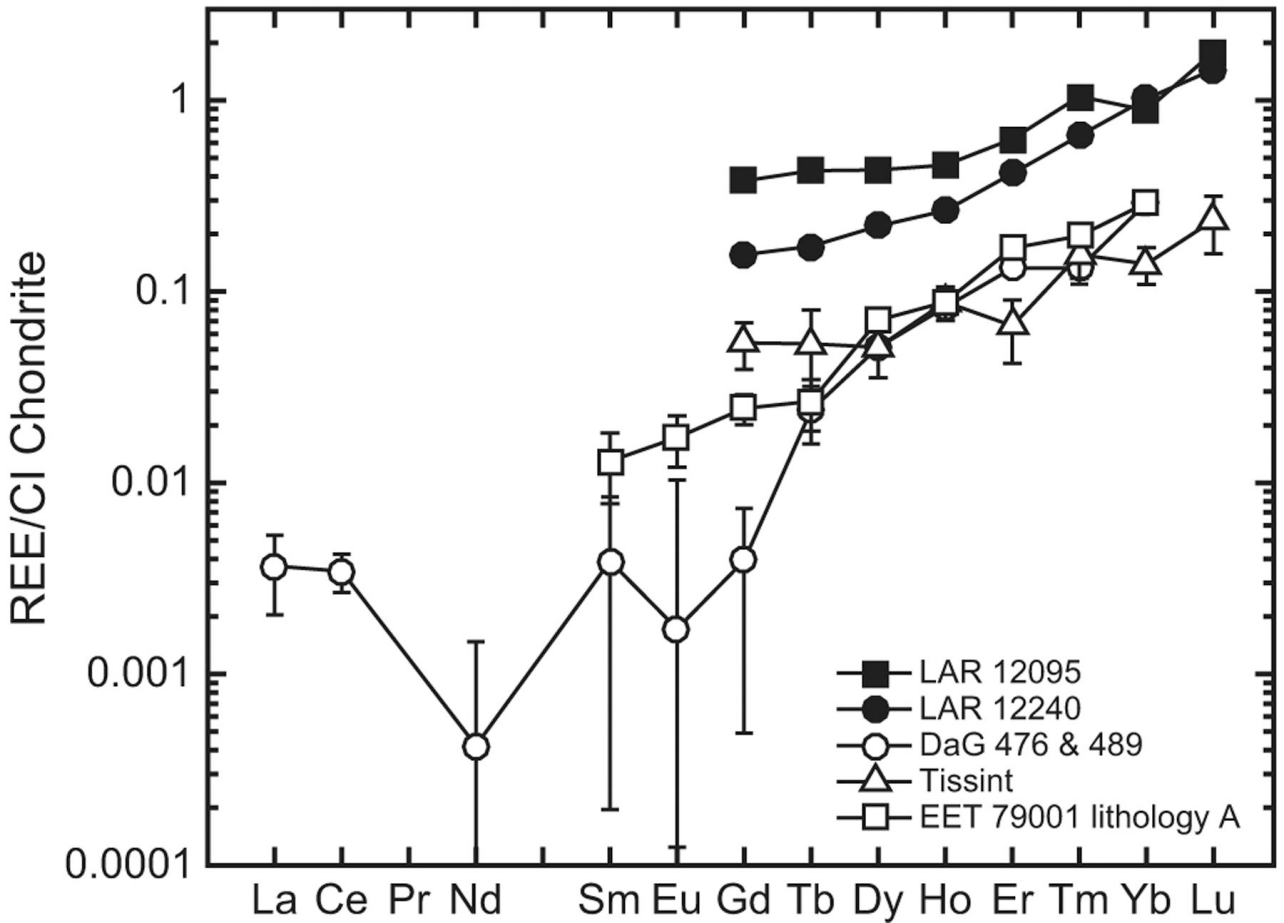
Representative REE abundances (normalized to CI chondrites; Palme et al. 2014) in olivine of LAR 12095 and LAR 12240 (filled symbols); only HREE abundances are shown since LREE contents were below the detection limit of our SIMS analyses. For comparison, REE abundances in olivine of the depleted olivine-phyric shergottites DaG 476 (Wadhwa et al. 2001), Tissint (Balta et al. 2015b), and the intermediate olivine-phyric shergottite EET 79001 lithology A (Wadhwa et al. 1994) are shown (open symbols). Error bars (±2σ) are uncertainties due to counting statistics only; in some cases, these are smaller than the symbols.

Since the REE abundances in olivines were low, we averaged the abundances in three olivine analyses obtained in each LAR 12095 and LAR 12240 thin section to obtain the representative REE concentrations (Table 3). This mineral has a strongly LREE-depleted pattern, with LREE abundances below the detection limit and CI-normalized Lu/Gd (or [Lu/Gd]_CI_) ~5 and ~9 in LAR 12095 and LAR 12240, respectively (Fig. 10).

#### Pyroxene

**Fig. 11.**
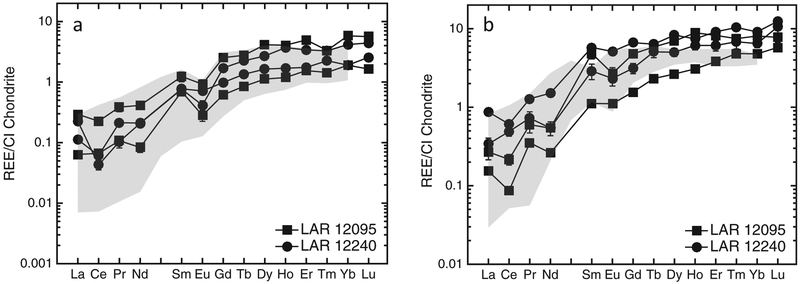
Representative REE abundances (normalized to CI chondrites; Palme et al. 2014) in the cores and rims of (a) low-Ca and (b) high-Ca pyroxene phenocrysts of LAR 12095 and LAR 12240. For comparison, shaded regions in (a) and (b) represent the compositional ranges of REE abundances in the low-Ca and high-Ca pyroxene of the depleted olivine-phyric shergottites DaG 476 and Tissint (Wadhwa et al. 2001; Balta et al. 2015b). Error bars (±2σ) are uncertainties due to counting statistics only; in some cases, these are smaller than the symbols.

A total of 13 measurements were made on pyroxenes in LAR 12095 (nine on low-Ca and four on high-Ca pyroxenes) and 15 in LAR 12240 (12 on low-Ca pyroxenes, which includes four analyses on the pyroxene inclusion within a pyroxene grain, and three on high-Ca pyroxenes). The REE patterns of pyroxenes in both LAR 12095 and LAR 12240 are LREE-depleted and similar in both samples (Fig. 11). The [La/Lu]_CI_ ratio for low-Ca pyroxenes (pigeonites) in LAR 12095 and LAR 12240 is ~0.04–0.06, while in high-Ca pyroxenes (augites) it is ~0.02–0.04. Moreover, this mineral is characterized by small to moderate negative Eu anomalies. In both samples, the CI-normalized Eu/Eu* (where Eu* is the interpolated value between CI-normalized Gd and Sm abundances) in pigeonites ranges from ~0.3 to ~1.0, and in augites ranges from ~0.6 to ~1.0. Most pyroxene analyses in these two shergottites (12 of the 13 in LAR 12095 and 9 of the 15 in LAR 12240) display negative Ce anomalies, i.e., with Ce/Ce* <0.75 per the convention used by previous studies (Floss and Crozaz 1991; Crozaz et al. 2003), where Ce* is the interpolated value between CI-normalized La and Pr abundances.

**Fig. 12.**
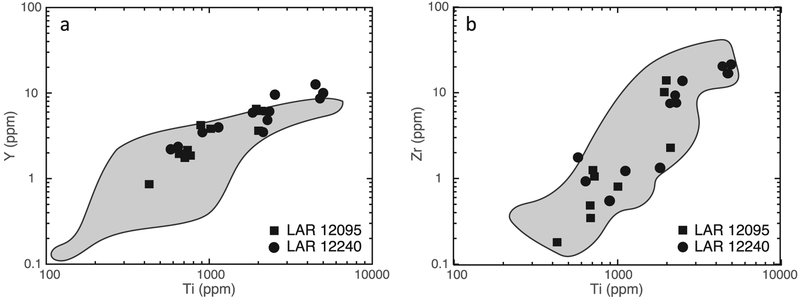
a) Y versus Ti, and (b) Zr versus Ti concentrations in low-Ca pyroxenes of LAR 12095 and LAR 12240. For comparison, shaded regions represent the compositional ranges of low-Ca pyroxenes of the depleted olivine-phyric shergottites DaG 476 and Tissint (Wadhwa et al. 2001; Balta et al. 2015b). Error bars (±2σ, due to counting statistics) are smaller than the symbols.

The REE abundances vary considerably in both pigeonites and augites in association with their major element compositions. The high-Mg pigeonite cores contain the lowest REE abundances among all the pyroxenes, with concentrations increasing with increasing Fe and Ca contents. Selected trace element (Ti, Y, and Zr) concentrations were measured in addition to the REEs in pyroxene grains in both samples. Figure 12 shows Y and Zr abundances plotted against Ti concentrations in pigeonites in LAR 12095 and LAR 12240.

**Fig. 13.**
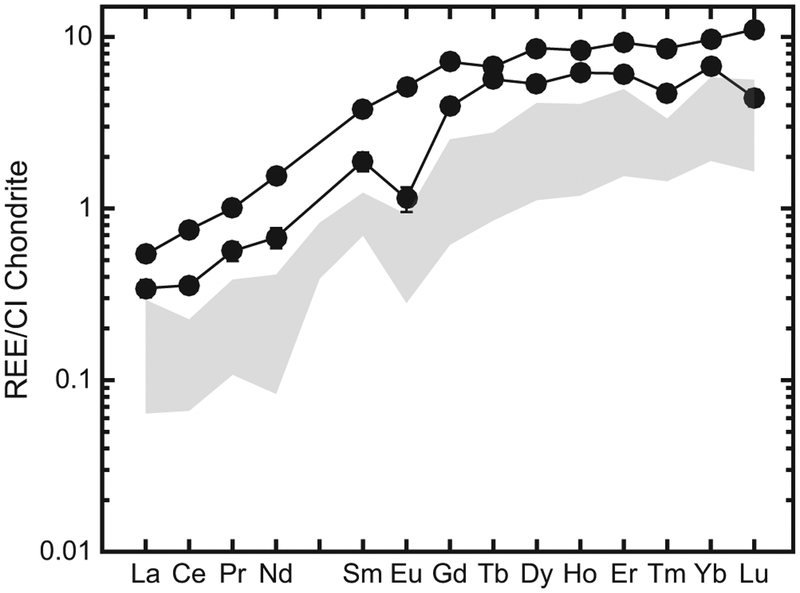
Representative REE abundances in the pyroxene inclusion from LAR 12240 shown in Fig. 4 normalized to CI chondrite abundances (Palme et al. 2014). For comparison, the shaded gray area is the range of REE abundances in other low-Ca pyroxenes of LAR 12095/12240 reported in Fig. 11. Error bars (±2σ) are uncertainties due to counting statistics only; in some cases, these are smaller than the symbols.

The range of REE abundances in 4 of the 12 low-Ca pyroxene analyses in LAR 12240 that were conducted on an inclusion (shown in Fig. 4) within a pigeonite phenocryst in this sample is shown in Fig. 13. As can be seen in this figure, REE abundances in this inclusion are higher than in the surrounding crystalline pigeonite. The sizes of the Eu anomalies in the four spots analyzed in this grain varied considerably, with Eu/Eu* ranging from ~0.4 to ~0.9. There are no Ce anomalies in any of these four analyses.

#### Maskelynite

**Fig. 14.**
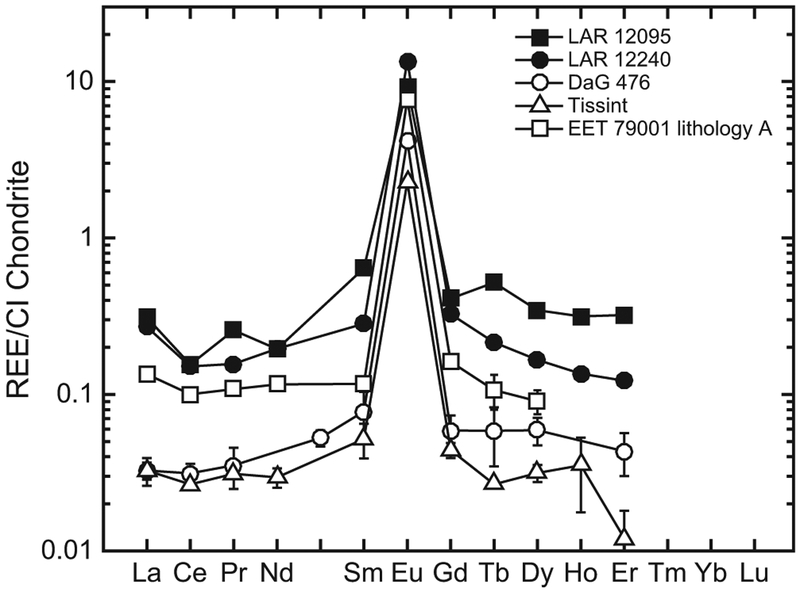
Representative REE abundances (normalized to CI chondrites; Palme et al. 2014) in maskelynite of LAR 12095 and LAR 12240 (filled symbols); HREE abundances beyond Er are not shown since these were below the detection limit of our SIMS analyses. For comparison, REE abundances in maskelynite of the depleted olivine-phyric shergottites DaG 476 (Wadhwa et al. 2001) and Tissint (Balta et al. 2015b), and the intermediate olivine-phyric shergottite EET 79001 lithology A (Wadhwa et al. 1994) are shown (open symbols). Error bars (±2σ) are uncertainties due to counting statistics only; in some cases, these are smaller than the symbols.

The abundances of the REEs were measured in three maskelynites in each of the sections of LAR 12095 and LAR 12240. This maskelynite phase in both samples exhibits a relatively flat LREE pattern, with [La/Sm]_CI_ ~0.5–1 and a large positive Eu anomaly (Eu/Eu* ~15–45) (Fig. 14). The HREE abundances are depleted (with Tm-Lu abundances below the SIMS detection limit). All maskelynite analyses in LAR 12095 and LAR 12240 showed negative Ce anomalies.

#### Merrillites

**Fig. 15.**
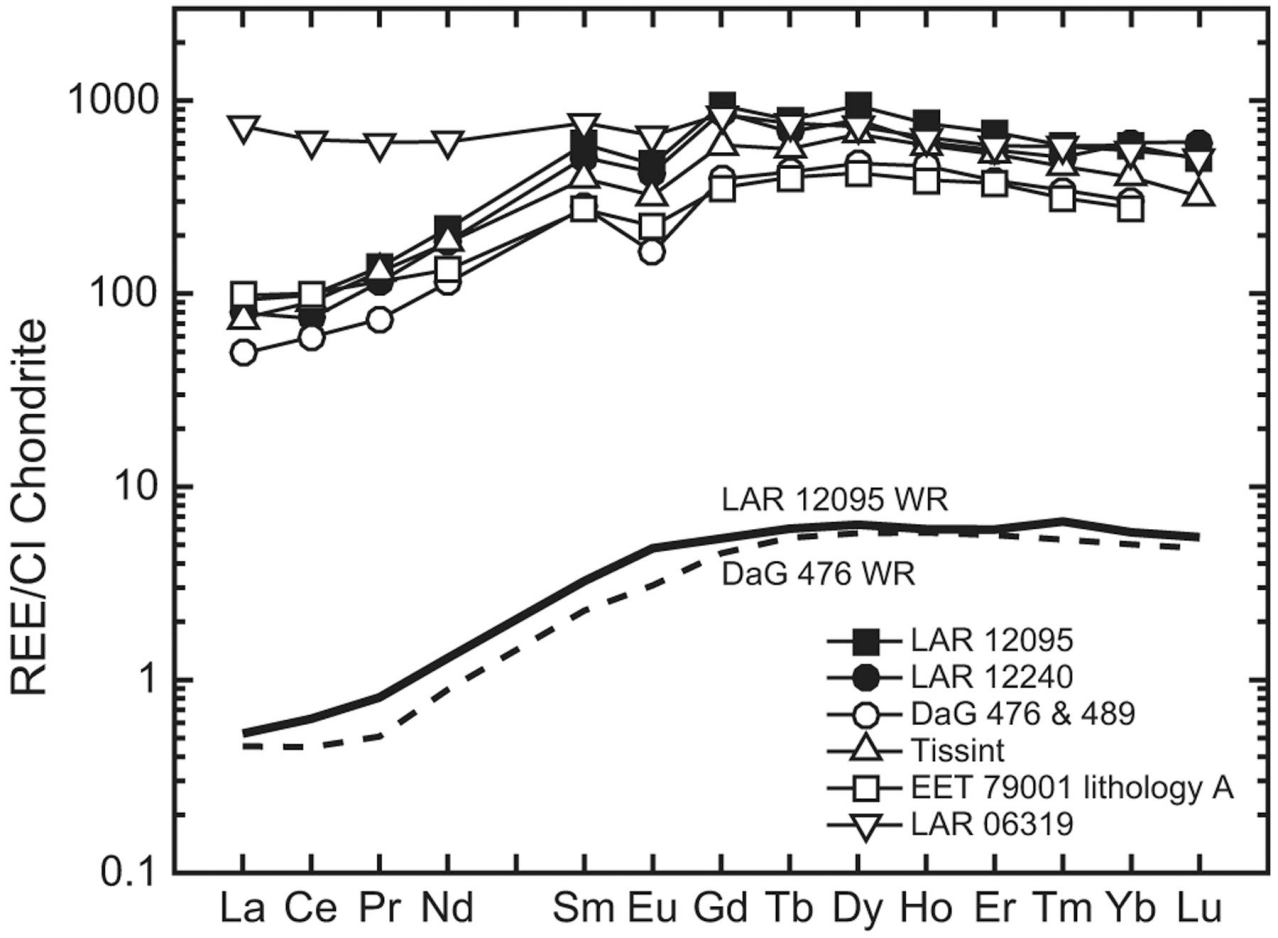
Representative REE abundances (normalized to CI chondrites; Palme et al. 2014) in merrillite of LAR 12095 and LAR 12240 (filled symbols). For comparison, REE abundances in merrillite of the depleted olivine-phyric shergottites DaG 476/489 (Wadhwa et al. 2001) and Tissint (Balta et al. 2015b), the intermediate olivine-phyric shergottite EET 79001 lithology A (Wadhwa et al. 1994), and the enriched olivine-phyric shergottite LAR 06319 (Balta et al. 2013) are shown (open symbols). Also shown here are the REE abundances in the whole rocks (WR) of LAR 12095 (Castle and Herd 2015) and DaG 476 (Wadhwa et al. 2001) as the solid and dashed black lines, respectively. Error bars (±2σ, due to counting statistics) are smaller than the symbols.

The REE abundances were measured in six merrillite grains in LAR 12095 and four in LAR 12240. Although this phosphate mineral is a minor phase, it is the major REE carrier in these samples and contains the highest REE concentrations of any measured phase (La ~80–100 × CI for LAR 12095 merrillite and La ~70–90 × CI for LAR 12240 merrillite). This mineral shows a LREE-depleted pattern ([La/Lu]_CI_ ~0.25 and ~0.15 in LAR 12095 and LAR 12240 merrillites, respectively), with a negative Eu anomaly (Eu/Eu* ~0.6–0.7 in both meteorites) (Fig. 15). With the exception of the Eu anomaly, the measured merrillite patterns are parallel to that of the LAR 12095 whole rock (Castle and Herd 2015) (Fig. 15).

## DISCUSSION

### Assessment of the Effects of Terrestrial Weathering

Recent work by Funk (2016) showed elevated S content in a band around the exterior surface of LAR 12095 that was absent from LAR 12240, which was used to suggest more extensive terrestrial alteration of LAR 12095 compared to LAR 12240. Our analyses also showed S-bearing phases, likely sulfates, intergrown with phosphate minerals only in LAR 12095 and not in LAR 12240, which could also be an indicator of a greater degree of weathering in LAR 12095. However, we note that phosphorus elemental maps showed high-P contents along cracks only in LAR 12240, not in LAR 12095 (Fig. 3); this P-enrichment along cracks is likely to be a result of terrestrial alteration in LAR 12240. As such, different indicators of terrestrial alteration appear to show somewhat different degrees of such alteration in these two samples. It is evident, however, that both paired samples have indeed suffered moderate to extensive alteration in the Antarctic environment.

The presence of Ce anomalies in meteoritic phases that typically have low REE abundances (such as pyroxenes and maskelynites) indicates terrestrial weathering processes (Crozaz and Wadhwa 2001; Crozaz et al. 2003). In the Antarctic environment in particular, the Ce^3+^ is oxidized to Ce^4+^, which is less mobile than the trivalent LREE; this results in Ce anomalies in individual minerals and even whole rocks due to the preferential mobilization of the LREE other than Ce (Crozaz et al. 2003). Highly shocked meteorites such as the shergottites are particularly prone to this type of alteration since shock-induced cracks and defects likely provide pathways for LREE mobilization. The fact that most pyroxene analyses in the LAR 12095 and LAR 12240 shergottites display negative Ce anomalies (12 of 13 and 9 of 15, respectively) suggests that both meteorites were weathered extensively during their residence in the Antarctic environment. Nevertheless, as also noted by previous studies (e.g., Crozaz et al. 2003), the HREE (including Y, which behaves similarly to the HREE) and high field strength elements such as Ti and Zr in meteoritic pyroxenes appear to be largely unaffected by such alteration. Furthermore, LREE and HREE abundances in more REE-enriched minerals such as phosphates do not appear to be affected by this alteration to any significant degree.

### Are LAR 12095 and LAR 12240 Paired?

Initial characterization of LAR 12095 and LAR 12240 suggested that these two samples were fragments of the same fall (Mikouchi and Takenouchi 2014; Balta et al. 2015a; Castle and Herd 2015; Funk et al. 2015; Howarth et al. 2015). We evaluate the pairing hypothesis in detail in the context of the data reported here.

The petrography and mineralogy of the two samples are similar. Phase modal abundances match to within 2% and the grain textures are similar. Olivine CSD plots show minor differences (Fig. 5), but olivine is particularly poorly sampled in LAR 12240 due to the relatively small area of this sample in our allocated thin section and the presence of coarse phenocrysts that dominated this sample area. The major element compositions of olivine, pyroxene, and maskelynite match within error (Table 1), and the modal abundances of these phases are also similar. The modal abundances and compositions of minor and trace phases match in both samples as well; apatite is rare, MgO/FeO contents of merrillites are similar, and both samples contain rare high-Ni pyrrhotites that are uncommon in Martian samples (Table 2). The minor element abundances of the pyroxenes in both samples define overlapping compositional ranges (Fig. 7); although the range of minor element compositions is slightly broader in LAR 12240 pyroxenes, this observation likely reflects the larger number of pyroxene analyses in that sample.

The abundances of trace elements such as Ti, Y, and Zr in LAR 12095 and LAR 12240 pyroxenes define similar trends (Fig. 12). The REE patterns and abundances in phases of LAR 12095 and LAR 12240 are generally similar (Figs. 10, 11, 14, and 15). However, as can be seen in Fig. 11, pyroxenes in LAR 12095 and LAR 12240 have slight differences in their ranges of absolute REE abundances. These minor differences are most likely a sampling artifact, i.e., the limited number of analyses of trace element abundances in pyroxenes reported here (see Rare Earth Element Compositions section above) are not likely to represent the full compositional range in this mineral of these two samples.

The LAR 12095 and LAR 12240 shergottites are similar in their petrological and geochemical characteristics. All mineralogical, major, and trace element measurements reported here support the hypothesis that these samples are paired, with only minor differences in chemistry between the two sample sections. Nevertheless, the analyses reported here do not suggest pairing with any other well-studied olivine-phyric shergottite (including another such shergottite, LAR 12011, that was also found during the 2012–2013 Antarctic field season). Thus, from here on we will treat the two thin sections studied here as constituting samples of the same meteorite.

### Calculated Parent Melt Composition and Crystallization Sequence

It is typically difficult to estimate the major element composition of magmas parental to Martian meteorites as most of these samples have an accumulated crystal component. Moreover, diffusive equilibration of major elements in early-crystallized olivine and pyroxene in these meteorites is fast compared to magmatic processes such that these elements are often redistributed (e.g., Chakraborty 1997; Cherniak and Dimanov 2010). Previous assessments of major element parental melt compositions of olivine-phyric shergottites have been based on the compositions of melt inclusions in olivines and on the assumption of equilibrium with measured olivine core compositions (Peslier et al. 2010; Filiberto and Dasgupta 2011). Olivines in LAR 12095 and LAR 12240 show strong evidence of disequilibria; while the cores of the largest olivine phenocrysts have Mg# as high as ~70, the groundmass olivines and rims of phenocrysts typically have Mg# 58–60. We did not measure major element whole rock compositions for LAR 12095 and LAR 12240 in this study, so we cannot estimate the olivine composition that would be in equilibrium with a LAR 12095/12240 bulk rock composition directly. Previous studies of other olivine-phyric shergottites suggest that the parent melts of samples containing 16–17% olivine (i.e., similar to modal abundance of olivine in LAR 12095/12240) should be in equilibrium with Mg# 78–82 (Musselwhite et al. 2006; Filiberto and Dasgupta 2011; Balta et al. 2015b). Therefore, either LAR 12095/12240 accumulated extra olivine so that a substantial portion of the olivine abundance represents xenocrysts or antecrysts, or the olivines in this sample have been altered by diffusion and are far from their initial composition. These arguments demonstrate that olivine major element compositions in LAR 12095 and LAR 12240 cannot be used directly to reconstruct the parental melt major element compositions through any simple assumptions based on equilibrium crystallization.

We can also compare the olivine and pyroxene major element compositions to test for equilibrium. The highest Mg# olivine and pyroxene cores are not in equilibrium because their Mg/Fe distribution coefficient (*K*_D_) is 1.11, while the equilibrium *K*_D_ should be 1.2 (Longhi and Pan 1989). This means the olivine cores are more Mg-rich than is required for equilibrium with the most Mg-rich pyroxenes. As noted above, we cannot reliably estimate the parent melt Mg content or Mg# because there is no way to verify that the Mg# 70 olivines represent the composition of the earliest-formed olivines and have not undergone diffusive re-equilibration. However, we can infer that the olivine phenocrysts must be the first major phase to crystallize, as they are too magnesian to be in equilibrium with the most magnesian pyroxene. Also, based on our modal abundances, LAR 12095/12240 contains more olivine than could be crystallized by a parental melt in equilibrium with Mg# 70 olivine, indicating early crystallization of olivine prior to pyroxene crystallization. This result, combined with inclusions of spinel within olivines, suggests the general crystallization sequence was spinel → olivine → pyroxene → plagioclase → merrillite.

The presence of inclusions of olivine and pyroxene in this sample is a complication to this general crystallization trend, and implications based to their occurrence will be discussed in the Inclusions in Olivines and Pyroxenes and Implications for Petrogenesis on Mars section. A portion of the olivine megacrysts could be either xenocrysts foreign to this melt or antecrysts formed and reprocessed in an active magmatic system, as is suggested for some previously studied olivine-phyric shergottites (Peslier et al. 2010; Balta et al. 2013, 2015b). Liu et al. (2016) argued against the interpretation that the high olivine abundance in Tissint required antecrysts in that sample, noting that elemental maps of olivine did not show irregular phosphorus-rich cores as might be expected for grains that underwent multiple processing stages. As shown in Fig. 3, we do observe an irregular phosphorus-rich olivine core in a LAR 12095 olivine. This observation suggests that this sample may have undergone multiple stages of olivine crystallization and therefore contains antecrystic or xenocrystic olivine. However, the relatively phosphorus-rich core has homogenized with the surrounding olivine in its major element composition. Even if there is some antecrystic or xenocrystic olivine in this sample, the Mg# of the intact large olivine grains supports the statement that the olivine began crystallization before pyroxene.

**Fig. 16.**
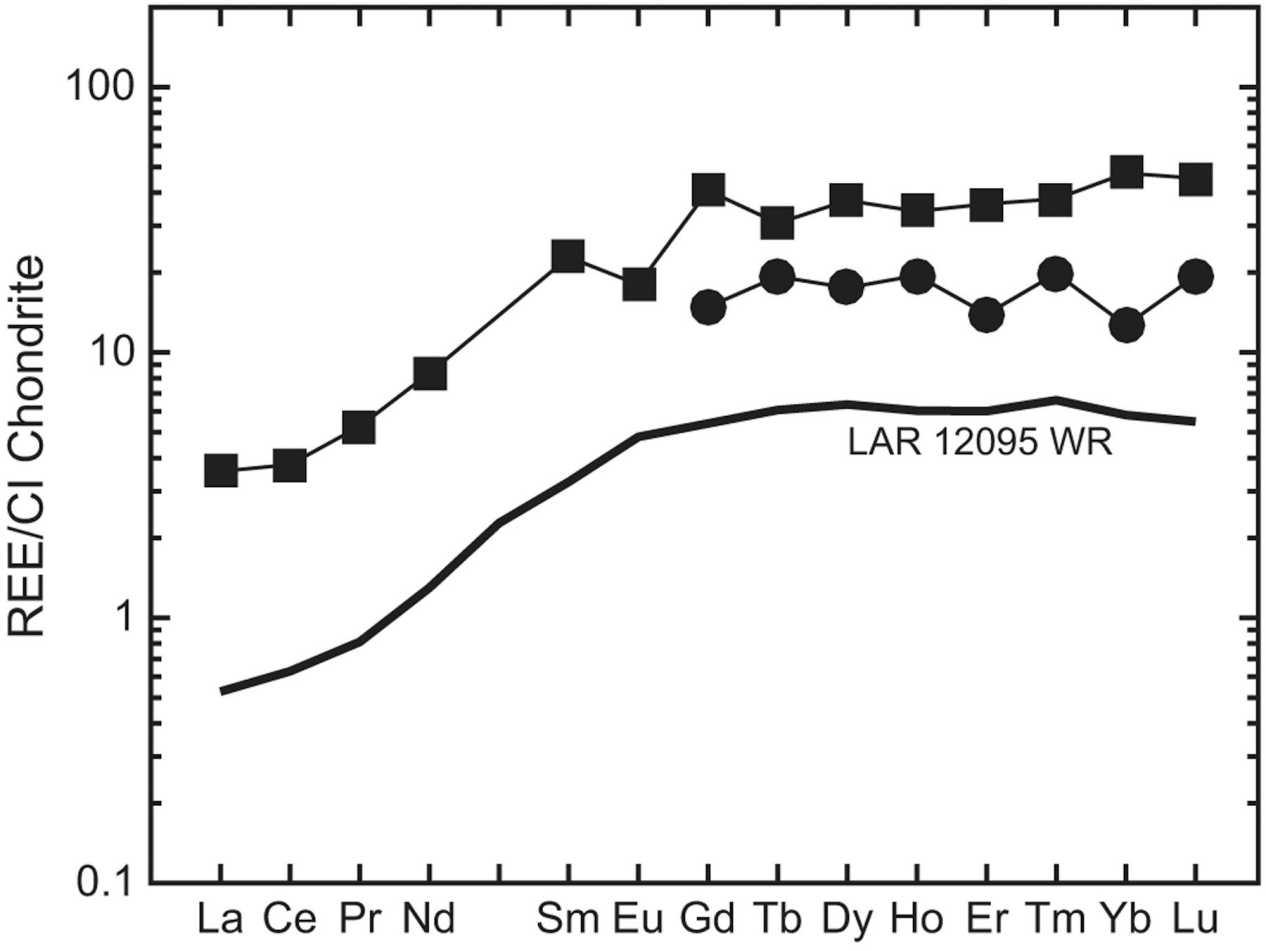
Calculated REE abundances (normalized to CI chondrites; Palme et al. 2014) in melts in equilibrium with the low-Ca pyroxene core (filled circles) and the merrillite (filled squares) in LAR 12095. The REE abundances in LAR 12095 whole rock (Castle and Herd 2015) are shown as the solid black line.

The REE abundances in the parental melt can be calculated using the measured abundances in the earliest formed REE-bearing minerals and appropriate partition coefficients. The REE composition of a melt in equilibrium with a specific phase is calculated with the equation C_PM_ = C_REE_/D_REE_, where C_REE_ is the concentration of the REE of interest in a given mineral, D_REE_ is the mineral/melt partition coefficient for that REE, and C_PM_ is the concentration of that REE in the parent melt. To estimate the REE abundances in the LAR 12095/12240 parent melt, we used the tabulated D_REE_ values for Low-Ca pyroxenes from Lundberg et al. (1990) that have been previously used to determine parental melt compositions in other shergottites (e.g., Balta et al. 2013, 2015b). Figure 16 shows our estimated HREE abundances in the LAR 12095/12240 parent melt calculated using the lowest REE concentrations measured in a low-Ca pyroxene core from LAR 12095 (presumably the earliest formed pyroxene in this sample). We could not make a robust estimate of the LREE abundances in the parent melt for two reasons, i.e., (1) the errors associated with the LREE partition coefficients for low-Ca pyroxene are up to ±50% given to the difficulty in determining these mineral/melt partition coefficients experimentally, and (2) as discussed previously in the Assessment of the Effects of Terrestrial Weathering section, the LREE abundances in most pyroxenes in LAR 12095/12240 appear to have been affected by terrestrial alteration. We note that the HREE pattern estimated for the LAR 12095/12240 parent melt is parallel to that of the LAR 12095 whole rock (Fig. 16).

Figure 16 also shows the estimated REE composition of the melt in equilibrium with a LAR 12095 merrillite is calculated using appropriate partition coefficients from Lundberg et al. (1990). As can be seen in this figure, the estimated REE pattern of this melt in equilibrium with one of the last crystallizing REE-bearing phases in LAR 12095/12240 closely parallels that of the whole rock. Furthermore, its HREE pattern is similar to the HREE pattern estimated for the LAR 12095/12240 parent melt. As is also the case for other shergottites (e.g., Wadhwa et al. 1994), the parallelism of the REE patterns of the melts estimated to be in equilibrium with the earliest-and latest-formed REE-bearing minerals with the whole rock REE pattern is indicative of progressive fractional crystallization in a closed-system following crystal accumulation.

### Magmatic Redox Conditions

It has been previously demonstrated that the magmatic *f*O_2_ of Martian basalts varies by 2–3 log units (Wadhwa 2001; Herd et al. 2002). As shown in these previous studies, these magmatic *f*O_2_ values appear to correlate with geochemical (e.g., whole rock La/Yb ratios) and isotopic systematics (e.g., initial ^87^Sr/^86^Sr and ^143^Nd/^144^Nd ratios). This could reflect heterogeneity in the shergottite source reservoirs in the Martian mantle or it may imply different degrees of mixing between reduced/geochemically depleted and oxidized/geochemically enriched silicate reservoirs on Mars. Here, we employ the mineral assemblage and Eu oxybarometer methods on LAR 12095 and LAR 12240 to estimate their magmatic redox state, thereby adding to the set of shergottite magmatic *f*O_2_ estimates to better understand redox reservoirs in the Martian mantle.

#### Redox Using Mineral Assemblage Oxybarometer/Major Elements

Castle and Herd (2015) utilized two geothermometers/oxybarometers (GTOB) to infer the *f*O_2_ of LAR 12095: the olivine spinel pyroxene (Ol-Sp-Px) GTOB and the titanomagnetite ilmentine (Tmt-Ilm) GTOB to represent early and late magmatic conditions, respectively. Their preliminary results (IW−0.1 ± 0.1 from Ol-Sp-Px and IW+2.8 ± 0.1 from Tmt-Ilm) suggest that the difference in oxygen fugacity between early and late magmatic redox is ~3 log units. Because this range exceeds the predicted range in a closed system, these authors suggested that LAR 12095 evolved as an open system, either involving addition or loss of material. However, these authors also noted difficulties associated with material addition, which is expected to have collateral effects on other chemical systems (Castle and Herd 2015; Herd 2003). Indeed, since the REE patterns of melts in equilibrium with the early-crystallized low-Ca pyroxene cores and late-crystallized merrillites are parallel with each other and with the whole rock REE pattern (see the Calculated Parent Melt Composition and Crystallization Sequence section above), any significant open system behavior involving addition of fluids or oxidized components into the melt is implausible. As an alternative, and as previously suggested for LAR 06319 (Balta et al. 2013), NWA 1183 (Shearer et al. 2013), and Tissint (Castle and Herd 2017), Castle and Herd (2015) and Shearer et al. (2013) suggested the possibility of material loss from the LAR 12095 parent melt by degassing of reduced components such as H_2_, H_2_S, Cl-bearing, or C-bearing species (such as CH_4_ or CO) as a means of altering the oxygen fugacity.

Our detailed mineral composition measurements can be used to estimate *f*O_2_ values. Using the range of major element compositions reported here of coexisting olivines, pigeonites, and spinels in LAR 12095/12240 and the calculator available at http://ctserver.ofm-research.org/Olv_Spn_Opx/index.php (Sack and Ghiorso 1989, 1991a, 1991b, 1994a, 1994b, 1994c), we calculated an *f*O_2_ estimate of IW+1.0 (FMQ−2.6). The typical error on the oxygen fugacity estimated previously for Martian meteorites with this technique is ±0.5 log units (e.g., Herd et al. 2001). Given this, our estimated *f*O_2_ value is somewhat higher than that reported by Castle and Herd (2015). However, we issue caution regarding the robustness of the values (obtained here and by Castle and Herd 2015) with the Ol-Sp-Px GTOB method for the LAR 12095/12240 shergottites as we believe it is unlikely that the associated minerals express a true equilibrium.

This calculator also estimates the equilibrium temperature between these minerals to be 931 °C. This temperature is far below the estimated magmatic temperatures for other depleted shergottites of ~1200 °C (Balta et al. 2015b). As noted previously, the olivine compositions we have reported here for LAR 12095/12240 are not in equilibrium with the most magnesian pyroxenes and, as such, do not represent the compositions of the first-crystallized olivines. Given the low calculated equilibrium temperature and our previous discussion of the potential resetting of the major element compositions of early-formed minerals by diffusion (see the Calculated Parent Melt Composition and Crystallization Sequence section), we expect systematic errors in the magmatic *f*O_2_ for LAR 12095/12240 calculated based on the Ol-Sp-Px GTOB. Due to the lack of mineral equilibrium in the early-crystallized phases, we have not attempted to address the hypothesis of Castle and Herd (2015) of increasing oxygen fugacity during crystallization. Rather, as discussed below, we have utilized an alternative oxybarometer based on Eu partitioning between low-Ca pyroxenes and melt to estimate the magmatic redox state to focus on estimating the oxygen fugacity of the early-formed pyroxenes.

#### Redox Using Eu Oxybarometer

While most REEs exist as trivalent ions in magmatic systems, europium can exist in a divalent or trivalent state depending on magmatic redox conditions. Specifically, the ratio of the mineral/melt partitioning of Eu relative to an adjacent REE (i.e., *D*_Eu_/*D*_Gd_ or *D*_Eu_/*D*_Sm_) for an early-formed mineral such as pyroxene is a function of the magmatic redox and can be used as an oxybarometer (e.g., McCanta et al. 2004). Wadhwa (2001) first applied this oxybarometer for determining the magmatic redox of various shergottites. Pyroxenes in shergottites are ideal for the application of this oxybarometer since they typically preserve igneous zonation of the REE due to fractional crystallization, such that their core compositions record early magmatic conditions.

We previously showed that the REE patterns of melts in equilibrium with early- and late-formed minerals in LAR 12095/12240 are parallel to that of the whole rock (Fig. 16) and are indicative of closed-system progressive fractional crystallization. As such, following Wadhwa (2001), it is assumed that the Eu/Gd ratio of the LAR 12095/12240 whole rock reflects that of its parent melt. Therefore, we used the whole rock composition for LAR 12095 reported by Castle and Herd (2015) along with the low-Ca pyroxene core composition for LAR 12095/12240 (i.e., pigeonite with the lowest REE concentrations in Table 3) to calculate a value for the pigeonite *D*_Eu_/*D*_Gd_ of 0.31 ± 0.13. Then utilizing the *D*_Eu_/*D*_Gd_ versus *f*O_2_ calibration curve for low-Ca pyroxene from McCanta et al. (2004), we estimate the magmatic fO_2_ for LAR 12095/12240 to be $\text{IW}-0.1_{-1.0}^{+0.8}$. The uncertainties in these calculations are propagated from 1σ errors (from counting statistics only) on the REE abundances in the low-Ca pyroxene. This estimate of the magmatic redox state for LAR 12095/12240 agrees with that obtained by Castle and Herd (2015) (IW−0.1 ± 0.1) using the Ol-Sp-Px GTOB. We also calculated the redox state at the time of formation of the high-Ca pyroxene using the Eu oxybarometer. For this calculation, we similarly used the whole rock abundances of Castle and Herd (2015) and the lowest REE abundances for core augite in Table 3 to calculate an augite *D*_Eu_/*D*_Gd_ of 0.8 ± 0.8. To obtain the redox conditions at the time of augite formation, we then used the augite calibration curve of Musselwhite and Jones (2002) to obtain a value of $\text{IW}+1.1_{-1.1}^{+1.1}$. This value is consistent, within the errors, with the magmatic redox state estimated at the time of formation of low-Ca pyroxene in LAR 12095/12240. When the errors in the redox estimates are considered, our calculations indicate that redox conditions did not evolve beyond ~1 log unit between the onset of crystallization of low- and high-Ca pyroxenes in LAR 12095/12240. These calculations also suggest that the redox state of the Martian mantle source of LAR 12095/12240 was close to ~IW, similar the source reservoirs of the other depleted olivine-phyric shergottites such as Dar al Gani (DaG) 476 and Elephant Moraine (EET) 79001 lithology A (Wadhwa 2001; Herd et al. 2002).

### The Effects of Post-Crystallization Shock Metamorphism

Due to the presence of the diaplectic plagioclase glass (maskelynite) in the LAR 12095 and LAR 12240 sections, their shock grade must be at least S4 (Stöffler et al. 2018). Both sections have localized shock features such as melt veins and melt pockets that are consistent with a shock grade ranging from S3 to S6. Funk et al. (2015) studied sections of LAR 12095 and LAR 12240 and noticed that both samples included shock melt veins. Since the focus of our study is on constraining the primary petrogenesis of LAR 12095/12240 on Mars, we did not specifically characterize the detailed mineralogy of the shock melt. Nevertheless, it is important to describe the shock features we observed in these paired olivine-phyric shergottite samples and place the process(es) that produced these in the context of the overall petrogenetic history of these shergottites.

We have observed several features that suggest that the paired LAR 12095 and LAR 12240 shergottites have experienced extensive shock. Similar to the findings of Funk et al. (2015), we found several long, thin shock melt veins (an example can be seen along the top edge of the stained olivine in Fig. 2a) in our studied thin sections. Our LAR 12095,22 section also showed evidence of localized melting associated with shear zones that cut larger grains. The largest shear zone offsets an olivine grain and is about 400 μm long (Fig. 3d). Along this shear zone is a small melt pocket <20 μm in width that contains sulfide blebs (Fig. 3e). A notable shock feature in the LAR 12240,16 section is an inclusion of poorly crystalline low-Ca pyroxene within a larger well-crystallized pigeonite grain; this inclusion is ~0.5 mm in diameter, appears dark and patchy in transmitted light and slightly lighter than surrounding pyroxenes in BSE (Figs. 4a–c). Many radial fractures emanate from the included grain (although few fractures penetrate it), suggesting that this region expanded more than the surrounding pigeonite during pressure release. Close inspection of the BSE image suggests that the BSE brightness near the center of the inclusion is similar to that of some of the surrounding pyroxenes; however, this inclusion contains rare sulfides (which explains the opacity of the feature in transmitted light) and possible minor olivine near the edge of this inclusion (identified only by increased BSE brightness). Another feature noticeable in the plane-polarized light images of the sections studied here (Figs. 1 and 2) is the dark, brown-tinted olivine. Takenouchi et al. (2018) studied this reddish-brown olivine in LAR 12095 as well as in other shergottites and concluded that such olivine is often accompanied by thin melt veins and is likely formed under conditions similar to those that produced melt veins and melt pockets (i.e., high shock pressure of ~55 GPa). Based on bulk rock features, such as the presence of maskelynite, the shock grade of LAR 12095 and LAR 12240 is at least S4. However, local shock features such as melt veins and melt pockets suggest that localized regions in these samples were shocked to higher degrees; nevertheless, these shock features do not appear to have affected the major or trace element compositions reported here of the individual phases (where measurements were made away from such features).

#### Comparison to Other Olivine-Phyric Shergottites

Many of the mineralogical, petrographic, and geochemical characteristics reported here for the LAR 12095/12240 pair suggest affinities to the depleted olivine-phyric shergottites such as DaG 476, Dhofar 019, Tissint, and Y-98. Major element compositions of various phases in LAR 12095/12240 are generally similar to those in other depleted shergottites, but some differences exist. Olivines are generally more Fe-rich in comparison to many other olivine-phyric shergottites. The most Mg-rich olivine cores we found have Mg# 70, which is among the lowest in olivine cores of olivine-phyric shergottites; this value is comparable to olivine cores in Dhofar 019 and NWA 1068 (Taylor et al. 2002; Barrat et al. 2002), although olivine rims and groundmass olivines in these samples are also significantly more Fe-rich than in LAR 12095/12240. The major element compositions of olivine rims and groundmass olivines in LAR 12095/12240 resemble those in DaG 476 and SaU 005/094. Thus, the total range in olivine major element compositions is the narrowest reported thus far for any olivine-phyric shergottite (e.g., Basu Sarbadhikari et al. 2009; Balta et al. [2015b] and references therein). The range in Mg# (70–58) and the common occurrence of Fe-rich olivines is similar to the description of the poikilitic shergottites RBT 04261 and NWA 7397 (Mikouchi and Kurihara 2008; Howarth et al. 2014); in both these shergottites, the most Mg-rich olivines are found in the cores of olivines included in pyroxene oikocrysts, while the low-Mg olivines are found in the nonpoikilitic regions. Pyroxenes in LAR 12095/12240 also define a narrow range in major element compositions and are similar to but slightly more Fe-rich than those in SaU 005/094 (Zipfel et al. 2000; Gnos et al. 2002), although they are slightly more Fe-rich. Augite and pigeonite major element compositions in LAR 12095/12240 also overlap with those in DaG 476 (Zipfel et al. 2000); unlike DaG 476, however, these samples lack orthopyroxene. Pyroxene major element compositions in poikilitic shergottites LEW 88516, ALH 77005, and NWA 7397 (Howarth et al. 2014; Harvey et al. 1993) are also generally similar to those in the LAR 12095/12240 pair. Maskelynite major element compositions in LAR 12095/12240 resemble those in SaU 005/094, although the latter are more Na-rich (Zipfel et al. 2000; Gnos et al. 2002). Phosphate Mg/Fe ratios in LAR 12095/12240 are similar to those in SaU 005/094 (Gnos et al. 2002). The large range of Ni contents in pyrrhotite of LAR 12095/12240 is similar to that in pyrrhotite of SaU 005/094 (Gnos et al. 2002), although the LAR 12095/12240 pair contains sulfides with a higher Ni content.

The LAR 12095/12240 CSD of olivine at coarse grain sizes generally falls within the range represented by other olivine-phyric shergottites, with similar concave-up/negative slope patterns. Olivine abundances in LAR 12095 and LAR 12240 are slightly lower than in Tissint, higher than in Y-98, and closest to those observed in LAR 06319.

The REE patterns in the whole rock (Castle and Herd 2015) and individual phases of the LAR 12095/12240 pair indicate that they are depleted olivine-phyric shergottites that crystallized in a magma reservoir under relatively reducing conditions (~IW). These characteristics are most comparable to DaG 476 (Wadhwa et al. 2001), Dhofar 019 (Taylor et al. 2002), Tissint (Balta et al. 2015b), SaU 005/094 (Dreibus et al. 2000; Gnos et al. 2002), and the intermediate shergottite EET 79001 lithology A (Wadhwa et al. 1994). However, REE abundances in phases of LAR 12095/12240 are higher than those in other depleted shergottites (Figs. 10–15). Olivine REE abundances in LAR 12095/12240 are about an order of magnitude or higher than those in DaG 476/489, SaU 005/094, and Tissint, although the slope of the HREE patterns in olivines of all these shergottites are similar. The ranges of low-Ca and high-Ca pyroxene REE abundances of LAR 12095/12240 overlap with those of DaG 489, EET 79001 lithology A, Tissint, and LAR 06319 (Wadhwa et al. 2001; Balta et al. 2013, 2015b). Since extensive terrestrial weathering of LAR 12095/12240 appears to have affected the LREE abundances in at least some of the minerals (typically those with relatively low REE abundances such as pyroxenes and maskelynites), it is more relevant to compare HREE abundances and patterns and Eu/Eu* ratios among minerals of the various depleted olivine-phyric shergottites. The pyroxenes of LAR 12095/12240 have generally comparable abundances and patterns of REEs to previously studied olivine-phyric shergottites (Fig. 11). Maskelynite REE patterns in LAR 12095/12240 most resemble those in the depleted olivine-phyric shergottites DaG 476 and Tissint and the intermediate olivine-phyric EET 79001 lithology A, although the positive Eu anomaly in LAR 12095/12240 maskelynite is slightly smaller (Eu/Eu* ~23–44) compared to maskelynites in the other three olivine-phyric shergottites (Eu/Eu* ~55–85), and REE abundances are somewhat higher (La × CI ~0.3) compared to maskelynites in the other three olivine-phyric shergottites (La × CI ~0.01–0.2) (Fig. 14). The REE patterns in LAR 12095/12240 merrillites are most similar to merrillites of DaG 476/489, Tissint, and EET 79001 lithology A, although the REE abundances are the highest in this mineral among these other olivine-phyric shergottites (Fig. 15). As is the case in other shergottites, merrillite dominates the REE budget. As such, the overall REE pattern of LAR 12095/12240 merrillite is quite similar to that of its whole rock (Fig. 15).

The trends defined by trace and minor element compositions (i.e., Y, Zr versus Ti abundances) in low-Ca pyroxenes in LAR 12095/12240 overlap with those defined by low-Ca pyroxenes in the depleted olivine-phyric shergottites DaG 476 and Tissint (Fig. 12). The overall range in abundances and positive correlations indicate that the parent magmas of these shergottites are compositionally related and evolved similarly during progressive fractional crystallization. Overall, when considering trace element (including REE) microdistributions in the various phases, LAR 12095/12240 appear quite similar to other depleted olivine-phyric shergottites, particularly DaG 476.

Our best estimate of the magmatic *f*O_2_ for LAR 12095/12240 (using the Eu oxybarometer) is $\text{IW}-0.1_{-1.0}^{+0.8}$. This value matches, within the errors, the magmatic redox estimated for several depleted shergottites including DaG 476, QUE 94201 (Wadhwa 2001), Dhofar 019 (Herd 2003), SaU 005/094 (Dreibus et al. 2000; Gnos et al. 2002), and Tissint (Balta et al. 2015b).

Previous studies have characterized the extensive shock in other known Martian meteorites, which can be compared to that recorded in LAR 12095/12240. Tissint includes shock veins with pyroxene, stishovite, ringwoodite, and olivine that is transformed to ringwoodite or dissociated to amorphous silicate plus magnesiowüstite (Baziotis et al. 2013; Walton et al. 2014). Yamato-000047, a poikilitic shergottite, contains a ~1.8 mm shock vein which is made of high-pressure pyroxene polymorphs and pyroxene glasses in a melt vein (Imae and Ikeda 2010). As described above, the depleted olivine-phyric shergottite DaG 476 is most similar to LAR 12095/12240, especially in their trace element microdistributions. The shock within DaG 735 (paired with DaG 476) is indicative of a high shock grade due to the presence of dissociated olivine (Miyahara et al. 2011). As described in The Effects of Post-Crystallization Shock Metamorphism section, the LAR 12095/12240 pair also experienced high shock (S6) given the presence of shear zones containing melt pockets and clasts, shocked poorly crystalline pyroxene surrounded by radial fractures, and majoritic garnet.

Finally, we note that there are currently no constraints on the crystallization age of LAR 12095/12240. However, assuming that these paired depleted olivine-phyric shergottites have a crystallization age in the range of ~400–550 Ma (similar to the ages determined for other depleted olivine-phyric shergottites like DaG 476, Dhofar 019, Tissint, and Y-98; Borg et al. 2001, 2003; Shih et al. 2005; Brennecka et al. 2014), Lu-Hf and Sm-Nd systematics in a whole rock sample were used to obtain model constraints on the isotopic characteristics of the mantle source reservoir of LAR 12095/12240 (Righter et al. 2015). This modeled source in the Martian mantle is most similar to that of DaG 476. Taken together, the petrographic and geochemical characteristics of LAR 12095/12240 imply that their mantle source and petrogenesis on Mars was similar to that of other depleted olivine-phyric shergottites, particularly DaG 476.

#### Inclusions in Olivines and Pyroxenes and Implications for Petrogenesis on Mars

The LAR 12095/12240 shergottites contain several examples of inclusions of one mineral in the same mineral, including a rounded inclusion of olivine within olivine (Fig. 3b) and a shocked pyroxene-composition inclusion within a pyroxene (Figs. 4a–c). Liu et al. (2013) reported inclusions of rounded to irregular olivine and pyroxene grains hosted as inclusions by larger grains of olivine and pyroxene in the EET 79001 lithology A shergottite. Those authors inferred that the most magnesian olivines and the rounded pyroxene inclusions were xenocrystic; the included minerals first formed at pressure when olivine and pyroxene could co-crystallize and were rounded due to remelting during adiabatic ascent to the surface prior to incorporation in newly grown minerals upon resumption of cooling.

Neither of the inclusions mentioned above in the LAR 12095/12240 samples provides as straightforward of an interpretation as those in EET 79001 lithology A, but together they may suggest similar processes. In EET 79001 lithology A, olivine inclusions were found inside pyroxene grains allowing a definitive identification of them as single-mineral inclusions, while in LAR 12095/12240 each inclusion is found only within the same mineral. Furthermore, there is no chemical discontinuity associated with the inclusion of olivine within olivine, making its identification in BSE impossible (Fig. 3). This lack of chemical discontinuity does not argue against this rounded inclusion predating the surrounding crystal, as diffusion in olivine is fast enough to homogenize a small inclusion like this one while the larger crystal was growing (Chakraborty 1997). We can better understand the origin of this inclusion in the context of the irregularly shaped high-P core in the large olivine grain (Fig. 3c). The high abundance of phosphorus in this core suggests the olivine grew rapidly and its location immediately adjacent to a melt inclusion suggests that this high-P core is xenocrystic or antecrystic. It likely formed with typical olivine morphology and developed irregular edges during a similar remelting event to the one that rounded the petrographically visible inclusion. After melting, this grain resumed growing and it trapped both the melt inclusion adjacent to the core and the rounded olivine inclusion. Therefore, the best explanation for the structure of this olivine is that olivine crystals formed and were rounded and eroded through remelting during either reheating or adiabatic ascent. The larger grain then resumed growing and the rounded inclusion was trapped inside. Finally, the long residence in that grain at magmatic temperatures homogenized the inclusion and surrounding grain.

The poorly crystallized pyroxene-composition inclusion in pyroxene is similarly complex to interpret because structural (Raman) analysis suggests this inclusion was heavily altered, and possibly melted, during shock. Chemical analyses of the included phases indicate that their major and minor element chemistry is closely related to other normal pyroxenes in this meteorite. For example, although the inclusion is REE-enriched relative to typical low-Ca pyroxene, the REE patterns parallel those of the low-Ca pyroxenes in LAR 12095/12240 (Fig. 13), indicating that these pyroxenes are not xenocrysts included in the sample. Although there are small inclusions within this pyroxene-composition inclusion that may be sulfide and possibly olivine (too small for accurate EPMA targeting), compositional analyses indicate that this inclusion also does not represent trapped melt because its major and minor element compositions lie on the same element evolution trends as other low-Ca pyroxenes in LAR 12095/12240 (Fig. 8).

Moreover, the Raman spectrum shows the characteristic peaks of pyroxene even though the magnitude of those peaks is substantially reduced compared to the crystalline pyroxene enclosing the inclusion (Fig. 9). The Raman spectrum clearly indicates that structurally the inclusion is no longer well-crystallized pyroxene, but it still retains enough of the characteristics of the pyroxene structure to suggest that it must originally have been pyroxene; we suggest that shock processes disrupted the original pyroxene structure of this inclusion. The radial fractures emanating from the inclusion indicate that it was included prior to the shock event and the minor phases (the smaller sulfides and olivine) may have been trapped in the pyroxene or could have exsolved out during the shock disruption. Like the inclusion of olivine within olivine, the edges of this inclusion are rounded; therefore, this pyroxene inclusion is consistent with pyroxenes formed during early crystallization and then partially remelted during reheating or decompression prior to structural disruption during shock. Shock-related structural disruption in shergottites is commonly associated with the conversion of plagioclase to maskelynite. Although not commonly observed in shergottite pyroxenes, the textural features in this inclusion are similar to the dense melt formation mechanism suggested for maskelynite (i.e., Chen and El Goresy 2000). Notably, the major element chemical composition of this inclusion both matches that of the surrounding pyroxene chemistry with no obvious shearing, and it is surrounded by expansion cracks (which likely occurred during relaxation of the dense glass).

The conclusion that both the olivine and pyroxene represent early-crystallized minerals that were partially remelted is consistent with the model for olivine and pyroxene inclusions described in detail by Liu et al. (2013) for EET 79001 lithology A. The presence of inclusions of both olivine and low-Ca pyroxene would indicate that both minerals must have been present or co-crystallizing, as suggested for EET 79001 lithology A. As noted previously, the olivine phenocryst cores are too magnesian to be in equilibrium with the most magnesian pyroxenes, so olivine and pyroxene coexisting would require that the inclusions either are xenocrystic or formed under elevated pressure conditions where olivine and pyroxene could co-crystallize. The pyroxene inclusion chemistry is nearly identical to the crystalline pyroxenes in the sample and the REE patterns are parallel, arguing against a xenocrystic origin for that inclusion. Therefore, as with the inclusions in EET 79001 lithology A minerals, the most likely origin for the inclusions in LAR 12095/12240 involves cocrystallization of the olivine and pyroxene at a pressure of ~1 GPa, close to the olivine-pyroxene multiple saturation pressure for most shergottite parental magmas (Musselwhite et al. 2006; Filiberto et al. 2010; Liu et al. 2013). Crystallization at elevated pressure would also explain the elevated REE abundances in the pyroxene-composition inclusion as REE partition coefficients can increase by up to an order of magnitude with ~1 GPa increase in pressure (Wood and Blundy 1997; Blundy et al. 1998). The D^REE^ increase required to account for the higher REE abundances in the pyroxene-composition inclusion is not unreasonable, although we cannot reliably estimate the pyroxene/melt partition coefficients for the crystallization conditions at 1 GPa given the uncertainty in the temperature without better constraints on the phase equilibria. The irregular, high-phosphorus core of the olivine shown in Fig. 3c may represent a result of similar high-pressure trapping followed by partial dissolution; the elevated REE contents of the olivines of LAR 12095/12240 compared to those in other olivine-phyric shergottites (Fig. 10) may also include a contribution from the formation of olivine cores at elevated pressures; however, this cannot be distinguished easily from the similar effects of shock melting.

As noted by Liu et al. (2013) for the EET 79001 lithology A olivine-phyric shergottite and Howarth et al. (2014) for the NWA 7397 poikilitic shergottite, this proposed trapping pressure is close to the base of the Martian crust (e.g., Wieczorek and Zuber 2004). However, the pyroxene-composition inclusion in a LAR 12240 pyroxene shows a range of Ti/Al ratios (crosses in Fig. 8f); many of these analyses have Ti/Al ratios consistent with relatively shallow crystallization (Fig. 8e), and yet this inclusion is found at the core of a low-Ca pyroxene grain (with high En content) having lower Ti/Al ratios suggestive of crystallization at higher pressure. It is possible that interaction of the magma with crustal rocks during ascent to the surface or crystallization of an aluminous mineral could contribute to the range of Ti/Al ratios found in the pyroxene-composition inclusion. However, there is no evidence for crustal assimilation recorded in our trace element measurements and plagioclase should not crystallize under these pressure conditions. The low Ti/Al ratios in the low-Ca pyroxene surrounding the inclusion (similar to those in experimental pyroxenes crystallized at ~1.6 GPa for a NWA 1068 starting composition; Filiberto et al. 2010), suggest that the pressure during crystallization of this pyroxene was far above the pressure estimated for the multiple saturation in EET 79001 lithology A, likely indicative of crystallization below the crust/mantle boundary on Mars. Under these conditions, most known shergottites would be expected to saturate in orthopyroxene only and thus would not produce the included olivines (e.g., Filiberto et al. 2010; Balta et al. 2013; Balta and McSween 2013) or any clinopyroxenes. It is surprising, then, that we observe augites with these low Ti/Al ratios (Figs. 8e and 8f). Thus, the pressures required by the Ti/Al ratios in pyroxenes of LAR 12095/12240 suggest that it is unlikely that trapping pressure alone is the controlling factor in the observed range of Ti/Al ratios in these pyroxenes. Filiberto et al. (2010) noted that it is possible for melt major element compositions to affect the pyroxene Ti/Al ratios. Other possible melt components, including volatiles such as water, have been shown experimentally to interact with Al solubility in pyroxene in terrestrial compositions, and could possibly represent one way to explain the measured pyroxene compositions (e.g., O’Leary et al. 2010). The high REE abundances may also reflect high solubility of Al in these pyroxenes at pressure (e.g., Blundy et al. 1998), so these pyroxenes may reflect a minor element solubility mechanism not active in the experiments of Filiberto et al. (2010). Overall, we suggest that the presence of olivine and phenocrystic pyroxene as inclusions in LAR 12095/12240 supports crystallization near the multiple saturation pressure of typical shergottites, close to proposed depths of the crust–mantle boundary as described for EET 79001 lithology A (Liu et al. 2013). However, we cannot definitively use the Ti/Al ratios of the pyroxene-composition inclusion to establish a trapping pressure for this inclusion in particular.

Trapping of the first-formed mineral inclusions in LAR 12095/12240 under crust–mantle boundary conditions could also be an additional explanation for the close chemical match between olivine and pyroxene in the LAR 12095/12240 pair and the poikilitic shergottites RBT 04261 and NWA 7397 (Mikouchi and Kurihara 2008; Howarth et al. 2014). The comparison between these two paired LAR shergottites and previously studied shergottite samples where crust–mantle boundary trapping has been argued suggests that trapping of melts at this depth may be a common physical process represented by multiple shergottite classes. Identification of similar mineral inclusions in other sections of these samples or in other shergottites may provide further constraints on magma storage and transport in the Martian crust.

## CONCLUSIONS

The olivine-phyric shergottites LAR 12095 and LAR 12240 are paired samples which are highly reduced and incompatible element depleted. This LAR 12095/12240 pair shares many petrogenetic details with other olivine-phyric shergottites. They show a standard crystallization sequence, with olivine and rare pyroxene phenocrysts forming early followed by plagioclase and minor and trace phases (oxides, sulfides, and phosphates) and likely magmatic redistribution of early-formed olivine and pyroxene phenocrysts. As is common with depleted shergottites, apatite is rare to absent (e.g., Balta et al. 2015a), and merrillite is the main phosphate mineral and the main REE carrier. The REE abundances and oxygen fugacity values are generally consistent with other depleted shergottites (Herd et al. 2002; Symes et al. 2008), and most closely match those of DaG 476 and Tissint. However, several distinct features of the LAR 12095/12240 pair set them aside from other shergottites. Specifically, LAR 12095/12240 have notably Mg-poor olivines compared with typical shergottites and a narrow range of olivine and pyroxene major element compositions, the REE abundances in olivine and maskelynite are notably high compared with those in similarly depleted shergottites, and they have low Al/Ti ratios in their pyroxenes. Finally, the LAR 12095/12240 paired samples contain rare inclusions of a mineral in the same mineral, i.e., an inclusion of olivine in olivine and a shocked, poorly crystallized pyroxene-composition inclusion trapped inside a well-crystallized pyroxene.

These analyses can be used together as a record of magmatic processes during LAR 12095/12240 petrogenesis even though there is evidence LAR 12095/12240 experienced terrestrial weathering. The LAR 12095/12240 pair crystallization process produced similar olivine size distributions to the olivine-phyric shergottite LAR 06319 along with evolved spinel and olivine compositions. The crystallization path followed closed-system progressive fractional crystallization as evidenced by trace element microdistributions in the various phases of LAR 12095/12240. The presence of inclusions of one mineral inside the same mineral suggests that the LAR 12095/12240 melt was trapped in the lower Martian crust prior to upwelling, remelting of early-formed phases, and final crystallization at shallow depths.

## Figures and Tables

**Table 1.** Representative compositions (in wt%) of major phases in LAR 12095 and LAR 12240.

	Olivine	Olivine		Pigeonite	Pigeonite	Pigeonite	Augite		Maskelynite	Maskelynite
*LAR 12095*										
SiO_2_	37.30	35.50	SiO_2_	53.13	52.24	52.20	50.97	SiO_2_	51.84	54.58
TiO_2_	0.02	0.02	TiO_2_	0.12	0.53	0.21	0.29	A1_2_O_3_	29.31	27.52
A1_2_6_3_	0.02	0.02	A1_2_O_3_	0.80	0.58	1.26	1.83	MgO	0.15	0.09
Cr_2_O_3_	0.07	0.05	Cr_2_O_3_	0.48	0.22	0.52	0.81	CaO	13.19	10.61
MgO	35.13	27.97	MgO	23.88	20.67	22.05	16.58	FeO	0.50	0.43
CaO	0.19	0.30	CaO	4.63	3.85	5.71	16.12	Na_2_O	3.83	5.09
MnO	0.50	0.63	MnO	0.56	0.62	0.54	0.41	K_2_O	0.08	0.10
FeO	25.91	34.98	FeO	15.73	20.71	16.73	11.53	Total	98.91	98.42
NiO	0.08	0.05	Na_2_O	0.06	0.04	0.06	0.18			
Total	99.26	99.75	Total	99.41	99.46	99.29	98.72			
Mg#	70.73	58.76								
*LAR 12240*										
SiO_2_	34.98	37.35	SiO_2_	53.31	52.10	52.09	52.27	SiO_2_	52.34	55.16
TiO_2_	0.04	0.00	TiO_2_	0.11	0.57	0.60	0.22	A1_2_O_3_	29.54	27.82
A1_2_O_3_	0.03	0.01	A1_2_O_3_	0.80	0.96	0.73	1.51	MgO	0.18	0.14
Cr_2_O_3_	0.21	0.07	Cr_2_O_3_	0.44	0.34	0.28	0.75	CaO	13.03	10.57
MgO	27.08	34.20	MgO	23.18	19.46	20.70	17.99	FeO	0.43	0.41
CaO	0.17	0.22	CaO	4.36	6.12	4.56	14.74	Na_2_O	3.73	5.05
MnO	0.62	0.52	MnO	0.55	0.59	0.68	0.42	K_2_O	0.08	0.15
FeO	35.31	27.11	FeO	16.41	19.31	20.36	10.87	Total	99.34	99.29
NiO	0.02	0.04	Na_2_O	0.06	0.09	0.09	0.16			
Total	99.17	99.54	Total	99.23	99.55	100.10	98.94			
Mg#	57.76	69.22								

**Table 2.** Representative compositions (in wt%) of minor phases in LAR 12095 and LAR 12240.

	Oxide	Oxide	Oxide		Sulfide	Sulfide		Merrillite	Merrillite
*LAR 12095*									
SiO_2_	0.33	0.07	0.07	Si	0.03	0.03	P_2_O_5_	44.87	44.99
TiO_2_	0.54	13.23	20.22	S	38.14	37.77	SiO_2_	0.10	0.10
A1_2_O_3_	8.06	5.89	4.40	Fe	58.61	55.14	SO_2_	1.07	0.34
V_2_O_3_	0.55	0.71	0.68	Co	0.00	0.00	A1_2_O_3_	0.08	0.12
Cr_2_O_3_	55.52	27.69	19.09	Ni	0.60	3.60	La_2_O_3_	0.00	0.02
MgO	3.17	2.44	3.87	P	0.01	0.00	Ce_2_O_3_	0.04	0.04
CaO	0.01	0.03	0.06	Mg	0.01	0.01	MgO	3.45	3.39
MnO	0.46	0.57	0.61	Al	0.01	0.00	CaO	46.60	46.55
FeO	30.96	46.83	49.07	Ti	0.00	0.03	MnO	0.07	0.06
NiO	0.00	0.01	0.09	Mn	0.02	0.00	FeO	1.05	1.19
Total	99.60	97.48	98.17	Ca	0.11	0.12	BaO	0.00	0.00
				Total	97.54	96.72	Na_2_O	1.53	1.35
							Total	98.85	99.99
*LAR 12240*									
SiO_2_	0.20	0.06	0.10	Si	0.02	0.03	P_2_O_5_	44.78	41.55
TiO_2_	0.62	16.00	20.22	S	38.73	37.10	SiO_2_	0.37	0.93
A1_2_O_3_	8.34	5.34	4.66	Fe	58.49	52.15	SO_2_	0.33	1.85
V_2_O_3_	0.51	0.71	0.73	Co	0.14	0.42	A1_2_O_3_	0.03	0.05
Cr_2_O_3_	55.13	27.75	16.02	Ni	0.59	7.43	La_2_O_3_	0.05	0.00
MgO	3.33	2.88	3.52	P	0.00	0.00	Ce_2_O_3_	0.03	0.00
CaO	0.01	0.03	0.16	Mg	0.01	0.01	MgO	3.39	3.72
MnO	0.43	0.56	0.53	Al	0.01	0.00	CaO	45.90	43.93
FeO	30.12	45.66	50.11	Ti	0.01	0.06	MnO	0.07	0.06
NiO	0.01	0.07	0.05	Mn	0.02	0.01	FeO	1.19	1.39
Total	98.69	99.05	96.09	Total	98.00	97.22	BaO	0.06	0.05
							Na_2_O	1.47	1.35
							Total	97.70	94.98

**Table 3.** Representative REE abundances (in ppm) in phases in LAR 12095 and LAR 12240.

	Low-Ca pyroxene		High-Ca pyroxene				
	Core	Rim	Core	Rim	Olivine	Maskelynite	Merrillite
*LAR 12095*							
La	0.015 ± 0.003	0.071 ± 0.007	0.037 ± 0.003	0.065 ± 0.014	b.d.	0.076 ± 0.005	22.69 ± 0.07
Ce	0.041 ± 0.003	0.140 ± 0.012	0.053 ± 0.004	0.134 ± 0.021	b.d.	0.099 ± 0.005	62.65 ± 0.14
Pr	0.010 ± 0.002	0.036 ± 0.006	0.033 ± 0.002	0.056 ± 0.012	b.d.	0.025 ± 0.001	13.19 ± 0.05
Nd	0.039 ± 0.010	0.195 ± 0.023	0.124 ± 0.008	0.256 ± 0.051	b.d.	0.092 ± 0.005	102.92 ± 0.26
Sm	0.107 ± 0.014	0.190 ± 0.035	0.171 ± 0.015	0.744 ±0.111	b.d.	0.099 ± 0.005	91.48 ± 0.98
Eu	0.017 ± 0.005	0.054 ± 0.010	0.065 ± 0.005	0.158 ± 0.029	b.d.	0.540 ± 0.012	27.17 ± 0.10
Gd	0.127 ± 0.012	0.524 ± 0.042	0.324 ± 0.020	0.988 ± 0.102	0.077 ± 0.003	0.084 ± 0.005	191.14 ± 0.35
Tb	0.032 ± 0.003	0.105 ± 0.011	0.088 ± 0.005	0.222 ± 0.025	0.016 ± 0.001	0.019 ± 0.001	29.93 ± 0.07
Dy	0.286 ± 0.014	1.056 ± 0.053	0.678 ± 0.030	1.781 ± 0.144	0.110 ± 0.004	0.088 ± 0.005	237.26 ± 0.50
Ho	0.067 ± 0.005	0.229 ± 0.019	0.174 ± 0.006	0.501 ± 0.039	0.026 ± 0.001	0.018 ± 0.001	42.42 ± 0.10
Er	0.256 ± 0.015	0.822 ± 0.043	0.629 ± 0.024	1.359 ± 0.117	0.104 ± 0.005	0.053 ± 0.003	114.40 ± 0.32
Tm	0.038 ± 0.003	0.087 ± 0.009	0.126 ± 0.005	0.195 ± 0.025	0.027 ± 0.001	b.d.	14.55 ± 0.06
Yb	0.320 ± 0.016	0.979 ± 0.060	0.806 ± 0.023	1.361 ± 0.116	0.147 ± 0.006	b.d.	86.98 ± 0.26
Lu	0.041 ± 0.004	0.141 ± 0.014	0.144 ± 0.006	0.194 ± 0.027	0.045 ± 0.002	b.d.	9.21 ± 0.04
*LAR 12240*							
La	0.054 ± 0.010	0.027 ± 0.002	0.210 ± 0.004	0.082 ± 0.016	b.d.	0.066 ± 0.004	19.44 ± 0.04
Ce	0.026 ± 0.002	0.038 ± 0.002	0.377 ± 0.005	0.304 ± 0.040	b.d.	0.096 ± 0.004	47.52 ± 0.08
Pr	0.010 ± 0.007	0.020 ± 0.001	0.120 ± 0.002	0.068 ± 0.014	b.d.	0.015 ± 0.001	11.04 ± 0.02
Nd	0.099 ± 0.008	0.100 ± 0.004	0.715 ± 0.010	0.257 ± 0.051	b.d.	0.093 ± 0.002	87.98 ± 0.15
Sm	0.117 ± 0.012	0.119 ± 0.007	0.886 ± 0.011	0.444 ± 0.100	b.d.	0.044 ± 0.001	78.08 ± 2.02
Eu	0.024 ± 0.005	0.042 ± 0.003	0.302 ± 0.003	0.137 ± 0.027	b.d.	0.784 ± 0.003	24.60 ± 0.05
Gd	0.354 ± 0.015	0.204 ± 0.009	1.372 ± 0.019	0.649 ± 0.095	0.032 ± 0.002	0.067 ± 0.001	175.77 ± 0.21
Tb	0.085 ± 0.003	0.051 ± 0.002	0.242 ± 0.005	0.193 ± 0.030	0.007 ± 0.000	0.008 ± 0.000	25.94 ± 0.05
Dy	0.689 ± 0.071	0.419 ± 0.015	2.118 ± 0.028	1.282 ± 0.148	0.056 ± 0.002	0.042 ± 0.001	200.22 ± 0.35
Ho	0.209 ± 0.021	0.096 ± 0.004	0.419 ± 0.008	0.346 ± 0.043	0.015 ± 0.001	0.008 ± 0.000	33.78 ± 0.06
Er	0.563 ± 0.073	0.289 ± 0.012	1.512 ± 0.025	1.012 ± 0.153	0.069 ± 0.002	0.020 ± 0.001	92.36 ± 0.19
Tm	0.086 ± 0.012	0.059 ± 0.002	0.270 ± 0.005	0.178 ± 0.033	0.017 ± 0.001	b.d.	12.60 ± 0.02
Yb	0.703 ± 0.059	0.321 ± 0.012	1.537 ± 0.027	1.090 ± 0.121	0.168 ± 0.005	b.d.	91.90 ± 0.12
Lu	0.111 ± 0.012	0.064 ± 0.003	0.311 ± 0.006	0.265 ± 0.055	0.037 ± 0.001	b.d.	13.42 ± 0.03

Errors on concentrations are 1σ from counting statistics only.

b.d. = below detection limit.
